# The study of aggression and affiliation motifs in bottlenose dolphins’ social networks

**DOI:** 10.1038/s41598-022-22071-w

**Published:** 2022-11-16

**Authors:** Ana Pérez-Manrique, Juan Fernández-Gracia, Antoni Gomila, José J. Ramasco

**Affiliations:** 1 Human Evolution and Cognition Research Group (EvoCog), Psychology Department, University of the Balearic Islands, 07122 Palma de Mallorca, Spain; 2 Instituto de Física Interdisciplinar y Sistemas Complejos IFISC (CSIC-UIB), Campus UIB, 07122 Palma de Mallorca, Spain

**Keywords:** Computational biophysics, Animal behaviour, Behavioural ecology

## Abstract

Networks in biology have provided a powerful tool to describe and study very complex biological processes and systems such as animal societies. Social network analysis allows us to assess different processes occurring in animal groups. In the current study, we use this approach to investigate how conflict dynamics and post-conflict interactions shape the social networks of groups of captive bottlenose dolphins. We first examined temporal changes and aggression-affiliation motifs in the observed dolphins’ network structure. Using the results of the previous analysis, we built two models that simulate the dynamics of aggression and affiliation in a small dolphin group. The first model is based only on the observed statistics of interactions, whereas the second includes post conflict memory effects as well. We found that the resulting social networks and their most common motifs matched the association patterns observed in wild and captive dolphins. Furthermore, the model with memory was able to capture the observed dynamics of this group of dolphins. Thus, our models suggest the presence and influence of post-conflict behaviors on the structure of captive dolphins’ social networks. Therefore, the network approach reveals as an effective method to define animal social networks and study animal sociality. Finally, this approach can have important applications in the management of animal populations in captive settings.

## Introduction

Living in groups provides survival benefits to social animals: protection, reduction of the risk of predation, increased reproductive fitness or higher probabilities to find resources^1,2^. Therefore, it is crucial to establish and maintain social relationships with other group members. Furthermore, affiliative and agonistic in-group interactions shape the hierarchy and social structure of the species. In species that rely on cooperation and mutual assistance for their survival, aggression might be constrained by the need to maintain social relationships as well as costs in terms of energy and risk^3–7^. Therefore, the reproductive and survival success of both opponents may depend on how conflicts are stopped^8^ and resolved^9^. Social species are then expected to develop ways to control and palliate the consequences of in-group aggression^3,4^. In fact, post-conflict management mechanisms are widespread among social species of primates, canids, or birds^7,10–13^. Active conflict resolution could mitigate the cost of conflicts and prevent further aggression through affiliative interactions that take place after an aggressive encounter^6^. After the end of the aggressive event, thus, former opponents may display a variety of interactions that may alleviate post-conflict distress, reduce aggressive tendencies in both parties, and restore relationships between former opponents^6^. Some of those post-conflict interactions are reconciliation (affiliative contacts between former combatants), third-party affiliation (affiliative contacts between one of the rivals and a bystander) or redirected aggression (aggressive behavior directed to a bystander by one of the former opponents)^11,14^.

Biological systems such as social groups of animals can be described as complex systems as long as they are composed of many components that interact with each other^15^. In behavioral ecology, this approach based on depicting animal groups as social networks, has been increasingly used in recent years. The social network approach could serve to study and understand the form and function of social relationships. It provides a way to study animal behavior in the context of the animal’s social environment. Moreover, using network analysis we can explore the emergence of patterns of behavior at the group and population level^16^.

Animal social networks are usually constituted by nodes representing individuals connected through a set of links that depict interactions between them, although nodes could represent more generally entities (like groups) and links could represent any type of relation among the entities. Links can be characterized by a binary variable (e.g., 0 or 1 indicating respectively the existence or not of an interaction) (unweighted network)^17^. Links can also be characterized by a number indicating the weight or strength of the interaction (weighted network)^17,18^. Furthermore, we can also distinguish between directed and undirected networks depending on whether their links have or not a direction indicating, respectively, a one-way or a two-way relationship. Moreover, in animal social networks we can also find signed networks in which the interactions between group members are described by a sign (+ or −) indicating positive (affiliative) or negative (agonistic) interactions respectively.

Focusing on the structure and function of certain network motifs has been a common way of studying social processes since these local interactions may link behaviors at the individual level with emergent network patterns^19^. Network motifs are particular patterns of interconnections between nodes that repeat themselves in one or several larger networks^20^. Examining aggressive and affiliative motifs in social networks can shed light on the mechanisms underlying the structure of the network. Furthermore, these motifs may indicate differences in functionality between networks^21^. For example, it has been found that social networks usually display bidirectionally-connected cliques (subset of a network in which the nodes are all connected to each other), which may indicate that individuals mutually strengthen relationships with their neighbors^20^. Several studies have assessed the impact of social conflict in network structure in different social species. For example, Dey et al. used the social network approach to study spatial associations and patterns of dominance interactions in captive social groups of a cooperatively breeding fish (*Neolamprologus pulcher*)^16^. Many other studies have assessed how affiliative networks are structured and their impact on different individual traits such as reproductive success or survival^19,22–27^. Nevertheless, the study of agonistic networks is much more restricted with only a few existing works examining the features of negative ties in animal social groups^17,23–25^.

Another interesting approach to animal sociality is the examination of temporal changes in social networks^26,28–30^. Several studies have analyzed how ecological variables such as food availability or seasonality shape the structure of social networks in primates^31,32^, cetaceans^33,34^ or elephants^35^. Nevertheless, our knowledge of how social procedures such as conflict influence animal social networks in time is scarce. Although aggressive and affiliative interactions among individuals of a social group are dynamic processes, most of the works on animal social networks consider static structures^28^. Animals might change their social interactions according to the result of previous contacts with other individuals.. Therefore, it is crucial to consider the temporal dynamics that influence animal social networks to better identify and understand the factors affecting animal sociality and the functions of social organization^28^. To date, there is only one study examining the impact of conflict on post-conflict social networks for both grooming and aggression networks in wild banded mongooses (*Mungos mungo*)^36^. This study focused on intergroup conflicts and it did contemplate post-conflict strategies*.* In contrast, our study aims to advance knowledge beyond static network studies assessing how conflict and post-conflict management strategies shape social networks in time.

In this work, we apply dynamic network tools to the study of conflict management in a group of captive dolphins. Bottlenose dolphins (*Tursiops truncatus*) are ideal candidates for the study of the dynamics of affiliative and aggressive interactions in social networks since they are a highly social species. They live in fission–fusion societies characterized by frequent variations in the composition of the group, flexible dominance relationships, and high levels of cooperation^37^. Despite the dynamic changes in the composition of the group, dolphins are dependent on their social partners establishing complex and stable relations with some group members^3^. Furthermore, conflicts are common among dolphins^38^, and several studies have shown that captive bottlenose dolphins display post-conflict mechanisms such as reconciliation and third-party affiliation^3,37,39,40^. For example, Yamamoto et al. showed that both, winners and losers, initiate reconciliation soon after the end of a conflict. These interactions decreased the probability of renewed aggression between former opponents and were more frequent between individuals sharing a strong bond^40^. Two studies also reported the occurrence of third-party affiliation in different groups of captive bottlenose dolphins^40,41^. These affiliative interactions reduced the probability of renewed aggressions suggesting that these contacts may also serve to ease tension^41^. However, the impact of ingroup aggression and post-conflict strategies on the structure and dynamics of the whole group has not yet been studied. Social network analysis can provide information on individual and group relationship changes after conflict and reveal how these changes affect the entire social network. Furthermore, there is a lack of studies applying dynamic modeling to assess responses of social networks to aggression and affiliative post-conflict interactions. An understanding of how social patterns emerge from these affiliative and agonistic interactions is crucial for making predictions regarding changes in social network structure in response to social conditions^42^. In addition, these dynamic models can provide information on the processes underlying social pattern formation and can generate social networks for quantitative predictions^42^.

This work seeks to assess the suitability of the network approach as a method to study conflict-related processes in animals, applying these methods to the study of dolphins’ post-conflict dynamics. With this general aim in mind, the following specific objectives were pursued:

1. Study the network architecture of groups of captive bottlenose dolphins using network motif analysis. Examining the most common affiliative and aggressive motifs of these networks can help us to better understand dolphins’ social dynamics. So far animal interactions have been studied separately, not relating affiliative with aggressive interactions. Using the social network approach, we aimed to address this important niche by studying affiliative and aggressive interactions in combination and from a dynamic point of view.
2. Examine how conflict and conflict resolution shape dolphins’ social networks in time. Using temporal networks allows us to study whether dolphins engage in post-conflict resolution interactions and assess the importance of these contacts in the social network structure. The use of dynamic networks instead of static ones has a great potential to provide insights into how changes in individual relationships could affect the entire social network and the dynamics of the whole group. Real social networks are dynamic systems in which interactions between group members are constantly shifting due to social or ecological variables. Therefore, a dynamic approach to animal social networks could reflect more accurately the real processes influencing the structure and dynamics of animal societies.
3. Apply to the study of animal conflict and post-conflict strategies the dynamic modeling approach. We aimed to derive generative models for temporal interaction data of the studied group of captive dolphins. In general, the social network approach in behavioral ecology has been focused on pattern analysis and there is a lack of dynamic interaction models applied to animal social groups^42^. Thus, with the construction of these models we try to go beyond traditional animal conflict studies and broaden our understanding of these dynamic processes. Furthermore, these models could help us understand and predict the responses of social networks to ingroup conflict in captive groups of animals. In this way, generative models could be a useful tool for managing animal populations in captive settings allowing animal caretakers to better select group composition to control ingroup aggression.

## Methods

### Subjects and facility

We observed two groups of Atlantic bottlenose dolphins (six different individuals in total) housed at the marine zoo “Marineland Mallorca”. One of the groups was composed of four individuals (G1) and the other was constituted by five individuals (G2). The two adult males and one of the females were the same in both groups (Table 1). Group composition changed due to the transfer of individuals to another pool of the zoo and due to the arrival of new individuals from another aquatic park.

**Table 1:** Age, sex, group, and identification number in the network of the subject dolphins. *M* male, *F* female.

Subject	Sex	Age (years)	Group and identification number in the network
Estel	F	13	G1: 1
Mateo	M	13	G1 and G2: 2
Blava	F	13	G1: 3 and G2: 1
Blue	M	25	G1 and G2: 4
Stella	F	8	G2: 3
Aitamy	M	7	G2: 5

The dolphins were kept in three outdoor interconnecting pools: the main performance pool (1.6 million liters of water), a medical pool (37.8 thousand liters of water) and a small pool (636.8 thousand liters of water). During the observational periods, the dolphins had free access to all the pools. Underwater viewing at the main and the small pool was available through the transparent walls around the rim of the pools.

### Ethics statement

This study was approved by the UIB Committee of Research Ethics and Marineland Mallorca. This research was conducted in compliance with the standards of the European Association of Zoos and Aquaria (EAZA). All subjects tested in this study were housed in Marineland Mallorca following the Directive 1999/22/EC on the keeping of animals in zoos. This study was strictly non-invasive and did not affect the welfare of dolphins.

### Behavioral observations and data collection

Behavioral data were collected in situ by APM from May to November 2016 for G1 and from November 2017 to February 2018 for G2. All observational periods were also recorded using two waterproof cameras SJCAM SJ4000. Observations were conducted at the main pool between 8:00 a.m. and 11:00 a.m. Due to the schedules and dynamics of the zoo, we were unable to collect data outside this period. Dolphin social behavior was registered and videotaped for 30 min–2 h each day. Only data from sessions that lasted at least 30 min were included in the analysis. We did not collect any data during training or medical procedures and resumed the observational session a few minutes after the end of these events.

We recorded all occurrences of affiliative and aggressive interactions, the identities of the involved individuals and the identity of the dolphin initiating the contact. Aggressive contacts were defined by the occurrence of chasing, biting, and hitting, as established in previous studies^37–41^. Affiliative contacts were defined as contact swimming, synchronous breathing and swimming (at least 30″ of continuous swimming) or flipper-rubbing, as established in previous studies^37,39–41,43^.

To assess the strength of the affiliative bonds in both groups, we calculated the index of affiliative relationships (IA) between dolphins following the procedure described in Yamamoto et al. For calculating the IA we recorded the relative frequencies of synchronous swimming since it is a well-defined affiliative behavior in dolphins. Data of synchronous swimming were recorded using group 0–1 sampling^44^ at 3-min intervals. This method consists of the observation of individuals during short periods and the recording of the occurrence (assigning to that period a 1) or non-occurrence (assigning to that period a 0) of a well-defined behavior^44^. For calculating the IA for each couple, the number of sampling periods in which synchronous swimming between individuals A and B occurred (X_AB_) was divided by the number of sampling periods in which individuals A and B were observed (Y_AB_): $IA=\frac{{X}_{AB}}{{Y}_{AB}}$^39,45^. Therefore, the IA reflects the level of affiliation for each dolphin dyad based on the pattern of synchronous swimming. This index served to construct the general affiliative social networks of both groups of dolphins.

### Temporal network construction

Temporal networks can provide insight into social events such as conflicts and post-conflict interactions in which the order of interactions and the timing is crucial. Furthermore, they allow us to calculate the probabilities of the different affiliative and aggressive interactions occurring in the group.

We used behavioral observations to construct temporal networks for each group. Each dolphin was treated as a node (N) with their aggressive and affiliative interactions supplying the network links. We divided the daily observations into periods of 3 min. In each period, we assigned a positive (+ 1), negative (− 1) or neutral (0) interaction to each pair of dolphins. That is, if during the period a pair of dolphins displayed affiliative interactions, we assigned a + 1 to the link between that pair of nodes, if they were involved in a conflict, we assigned a − 1, and if the pair did not engage in any interaction, we assigned to that link a 0. If during the same period, the pair displayed both aggressive and affiliative interactions we considered the last observed interaction. Therefore, we obtained an adjacency matrix (an N × N matrix describing the links in the network) for each group of dolphins. Thus, for each day we had a series of different signed networks of the group, each network representing a 3-min period.

### Social network analysis: time-aggregated networks and network motifs

We collapsed the temporal networks of each day in time-aggregated networks. This procedure consists in aggregating the data collected over time within specific intervals to create weighted networks. The sign and the weight of the links characterize these networks, indicating the valence and duration of the interaction respectively. Thus, they are static representations of the social structure of the group of dolphins. To obtain these time-aggregated networks we proceeded as follows:

First, for each day we aggregated the values of each interaction of the temporal networks until one link qualitatively changed. We considered a qualitative change if one interaction passed from being negative (− 1) to positive (+ 1) meaning that the pair of dolphins reconciled after the conflict or vice versa, or if a new affiliation (+ 1) or aggression (− 1) took place, that is the link changed from being neutral (0) to positive or negative. If a link changed from being negative or positive to being neutral, we did not consider that this interaction has changed qualitatively. For example, if dolphins interacted positively during two periods of time, then they ceased to interact (neutral) and finally they engaged in an aggressive interaction, the total weight of the interaction in the resulting time-aggregated network would be of + 2. Therefore, a conflict or an affiliation may extend over multiple periods containing several contacts, and is considered finished when the interaction changes its valence. In this way, we obtained a series of time-aggregated networks for each day, which retain the information on the duration, timing, and ordering of the affiliative and aggressive events in the group.

We examined the local-scale structure of the affiliative-aggressive social networks using motif analysis. Thus, for each group, we analyzed the network motif representation of the temporal and time-aggregated networks, identifying and recording the number of occurrences of each motif.

### Model of affiliative and aggressive interactions

We built two models (a simple and a complex one) that aim to simulate the dynamics of aggressive and affiliative interactions of a group of four dolphins. These models were created using the observed probabilities of each affiliative or aggressive interaction between individuals in group G1. We only used the data of G1 since we had more hours of video recordings and, thus, more statistics of the pattern of dolphins’ interactions. Both models return affiliative/aggressive temporal networks constituted by four nodes and different aggressive, affiliative, or neutral interactions between the six possible pairs of individuals in the network. We simulated data for 20 periods of 3 min per day for a total of 80 days to mimic the empirical data time structure. We obtained one temporal network for each period (1600 temporal networks in total) and ran 100 realizations of each model.

Our models work as follows: At the beginning of the simulations, all the interactions between the four nodes are neutral (0). In each period, we select a pair of nodes randomly and assign to that link a positive (+ 1) or a negative (− 1) interaction with probability *p* (calculated previously for each type of interaction). These interactions correspond to spontaneous aggressions and affiliations. In the complex model, if in the previous period a conflict took place, before assessing spontaneous interactions we first evaluated the different possible post-conflict contacts that could occur (reconciliation, new aggressions, and affiliations). Therefore, for reconciliations, we change the valence of the interaction from negative to positive with a certain probability. Then, we also randomly choose a pair of nodes including one of the former opponents and assign to that link a positive or negative interaction with the observed probabilities to simulate the occurrence of new affiliations (third party-affiliation) or redirected aggressions arising from the previous conflict. We keep on doing this procedure period by period. Lastly, we obtained the time-aggregated networks for the two models.

The simpler model only includes the probability of aggression and affiliation between group members, whereas the complex one also includes the patterns of conflict resolution previously observed. In this way, the complex model serves to assess the influence of post-conflict management mechanisms on the observed pattern of aggressive/affiliative networks. That is, the complex model also keeps track of past actions. Thus, depending on the interaction of the previous step, the probability of the following interaction changes based on the observed pattern of conflict resolution strategies.

### Calculation of the observed probabilities of affiliative and aggressive interactions

For the simple model, we calculated the probability of general aggression and affiliation per day without distinguishing between types of positive and negative interactions. Thus, we obtained the number of periods in which an aggressive or affiliative contact took place per day and divided it by the total number of periods of that day (probability of general aggression or affiliation per 3-min period). With these probabilities, we calculated the mean probability of general aggression and affiliation per period.

For the complex model, we calculated the probabilities of reconciliation, new affiliations/aggressions, and spontaneous affiliations/aggressions per day. That is, the probability that former opponents exchange affiliative contacts after an aggressive encounter (reconciliation), the probabilities that a conflict may promote new affiliations (third-party affiliation) or new conflicts (redirected aggression) between one of the opponents and a bystander in the same day, and the probability of affiliative or aggressive interactions not derived from a previous conflict (spontaneous interactions). To classify affiliations and aggressions in these categories we used the temporal networks, examining the interactions that took place after a conflict between opponents and between them and bystanders. If the opponents reconciled or affiliated with a bystander after a fight, we assumed that the following affiliative or aggressive interactions were spontaneous and were not a consequence of that conflict. Thus, to calculate the number of spontaneous affiliations, we subtracted the number of reconciliations and new affiliations from the total number of affiliations per day. For spontaneous aggressions, we subtracted the number of new aggressions to the total number of aggressions per day. Then, we obtained the probability of spontaneous affiliation and aggression per period.

Using the previous probabilities, we obtained the rate (r) of reconciliation, new aggression and new affiliation per minute with the following formula:${p=1-e}^{-r\Delta t}$. Using the same formula, we finally calculated the probability of reconciliation, new aggression and affiliation per 3-min period used in the complex model (Supplementary Table 1 for details of probabilities calculation).

### Network-motif analysis

We also carried out a network-motif analysis. As we did not consider the identities or sex of the nodes in these models, we grouped the obtained motifs into equivalent categories considering the pattern of interactions between nodes. We also classified the motifs obtained from the real data of G1 into those equivalent categories. Finally, we compared the pattern of equivalent network motifs of the observed social network of dolphins and the ones of the two models. To do so we calculated the Spearman’s rank correlation coefficient (*r*_*s*_), defined as a nonparametric measure of the statistical dependence between the rankings of two variables: ${r}_{s}=\frac{cov\left({rg}_{X}{rg}_{Y}\right)}{{\sigma }_{{rg}_{X}}}{\sigma }_{{rg}_{Y}}$; *rg*_*X*_ and *rg*_*Y*_ are the rank variables; *cov *(*rg*_*X*_* rg*_*Y*_) is the covariance of the rank variables, and *σrg*_*X*_ and *σrg*_*Y*_ are the standard deviations of the rank variables. Therefore, this coefficient allows us to assess the statistical dependence between the motif ranking of the real data and the one of each model.

### Computational implementations

All the models, network construction, visualization and motif analysis were generated and implemented using MATLAB R2018b.

## Results

### Empirical network analysis

A total of 217 affiliations and 133 conflicts were registered in G1 during the 80 days of recordings. In G2, a total of 91 affiliations and 44 conflicts were collected during the 23 days of recordings. The number of the different types of affiliations and aggressions recorded was, for G1: 41 reconciliations, 35 new affiliations, 141 spontaneous affiliations, 41 new aggressions, and 92 spontaneous aggressions. For G2: 16 reconciliations, 17 new affiliations, 58 spontaneous affiliations, 14 new aggressions, and 30 spontaneous aggressions.

**Figure 1:**
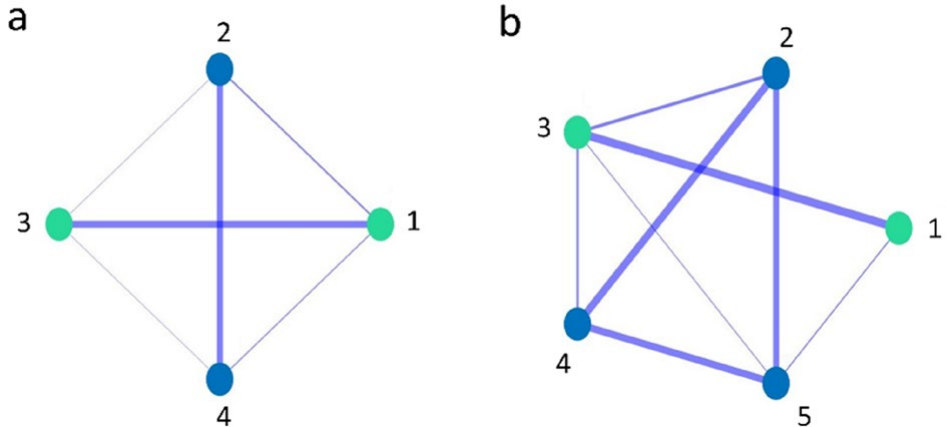
(**a**) Weighted social networks of G1 and (**b**) of G2. The width of the link represents the strength of the affiliative bond between individuals (IA). Green nodes represent females and blue ones, males.

Table 2 shows the IA for each pair of both groups of dolphins. The IA registered in these groups of captive dolphins showed that affiliation was higher among same-sex members in both groups (Table 3), although we cannot assess if this result depends on the specific identities and life histories of the animals, as we do not have enough statistics. Interestingly, in the group of five dolphins, the pattern of affiliative relationships is slightly different, reflected in a higher IA between males and females compared to that of G1 (Fig. 1).

**Table 2:** Index of affiliative relationships for each pair of dolphins.

Dyad	Group 1 network code	Group 2 network code	Group 1 IA	Group 2 IA
Estel-Mateo	1–2		0.116	
Estel-Blava	1–3		0.434	
Estel-Blue	1–4		0.079	
Mateo-Blava	2–3	2–1	0.050	0.000
Mateo-Blue	2–4	2–4	0.474	0.525
Blava-Blue	3–4	1–4	0.001	0.000
Blava-Stella		1–3		0.533
Blava-Aitamy		1–5		0.095
Mateo-Stella		2–3		0.214
Mateo-Aitamy		2–5		0.468
Stella-Blue		3–4		0.152
Stella-Aitamy		3–5		0.069
Blue-Aitamy		4–5		0.461

**Table 3:** Mean and standard deviation (SD) of the index of affiliative relationships for each possible combination of sexes in dolphin pairs. *F* female, *M* male. For female–female interactions we only have one interaction per group and thus we do not give any standard deviation. The same happens for male–male interactions in group G1.

Dyad	IA Group 1	IA Group 2	Total IA
M–M	0*.*474	0*.*485 ± 0*.*029	0*.*482 ± 0*.*025
F–M	0*.*061 ± 0*.*042	0*.*088 ± 0*.*077	0*.*078 ± 0*.*067
F–F	0*.*434	0*.*533	0*.*483 ± 0*.*049

The temporal networks revealed that, in both groups, affiliative contacts (G1: 1305; G2: 508) were much more numerous than aggressive ones (G1: 638; G2: 100). In both groups, the most numerous aggressive contacts were the ones between male–female pairs (G1: 571; G2: 73). In G1, the aggressions between male–female pairs even exceeded the number of affiliative contacts (G1: 318).

**Figure 2:**
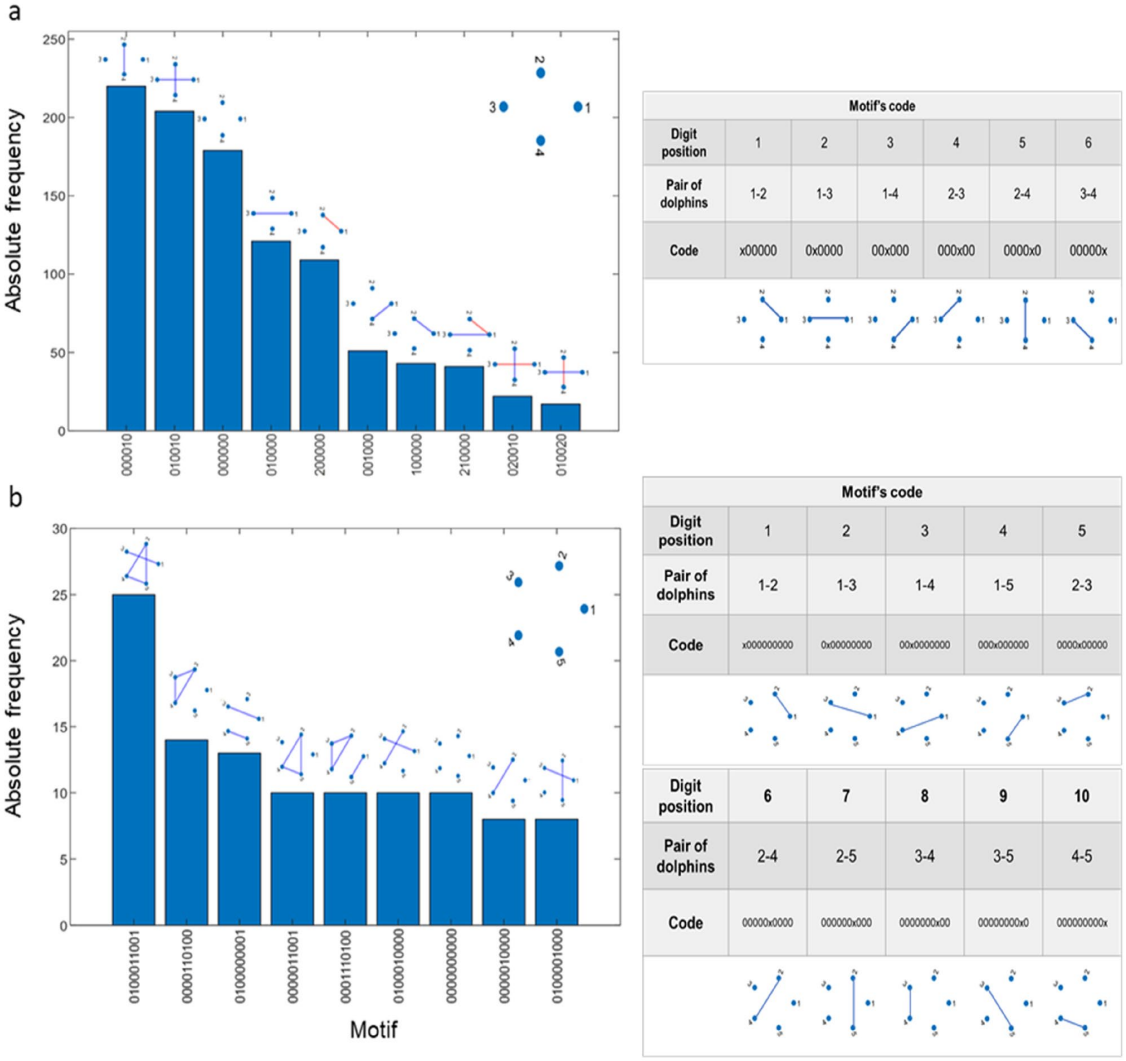
(**a**) Most common motifs in the temporal networks of G1 and (**b**) of G2. Red links represent aggressive interactions and blue links, affiliative interactions. No-interactions = 0, affiliative interactions = 1 and negative interactions = 2. The motif’s code is explained in the accompanying tables, specifying the digit position of each pair of dolphins and its location in the corresponding graph.

We show a histogram of the most frequent motifs for both groups of dolphins in Fig. 2. Motif analysis revealed that, in the group of four dolphins, the most common motif was a dyadic one including only a positive link between males. The second motif was the one with the two possible same-sex positive links (females and males engaged in affiliations with their same-sex partner at the same time) and was followed by a no-link tetrad and a motif with a positive link between females (Fig. 2a).

In the group of five dolphins, the most common motifs were also affiliative ones (Fig. 2b). The first motif was composed of four positive links: one link between females and the other three forming a closed triad between males. Then we found a triangle of positive links between one of the females and the two adult males, a motif composed of two positive links (one between the female pair and the other between a male pair), and a close triad of positive links between the three males.

In general, in the time-aggregated networks of both groups, positive interactions between individuals of the same sex were again the links presenting a greater weight. Thus, the longer affiliative interactions took place between same-sex pairs for both females and males (mean ± SD weight of positive links: 4.43 ± 4.23 for G1 and 4.13 ± 3.06 for G2). Nevertheless, sex will be omitted in the modeling, as we cannot assess that this is a general pattern due to the small number of individuals in both groups. On the other hand, aggressive interactions were shorter than affiliative ones as reflected by the lower weights of the negative links (2.74 ± 2.76 for G1 and 2.07 ± 1.60 for G2).

### Model of affiliative and aggressive interactions

The probabilities of affiliative and aggressive interactions per 3-min period were: general affiliation = 0.17 and general aggression = 0.07 for the simple model. For the complex model: spontaneous affiliation = 0.05, spontaneous aggression = 0.02, reconciliation = 0.04, new affiliation = 0.01, and new aggression = 0.01.

**Figure 3:**
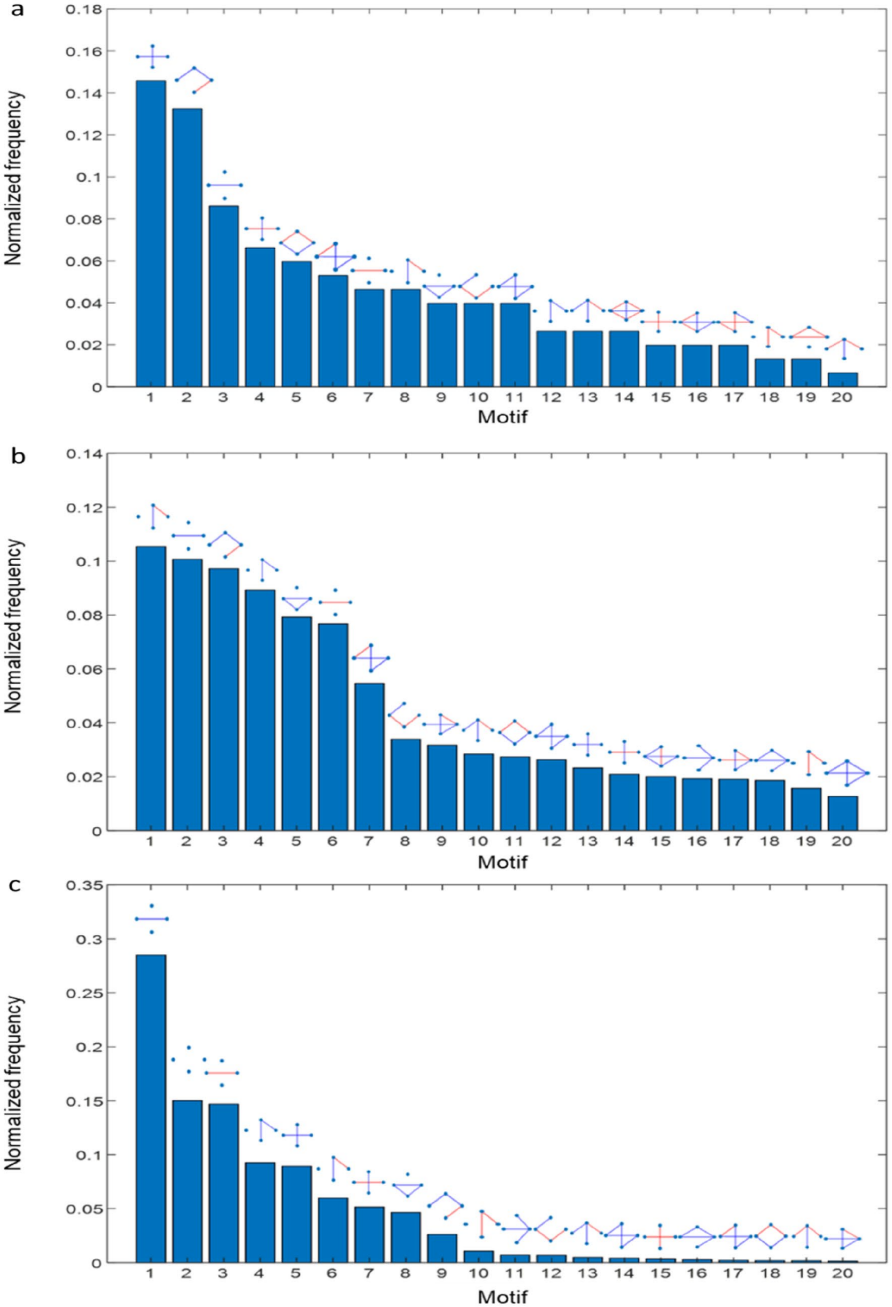
(**a**) Normalized histogram of the twenty most common network motifs in the time-aggregated networks of the real data, (**b**) of the simple model, and (**c**) of the complex model. Red links represent aggressive interactions and blue links, affiliative interactions.

**Figure 4:**
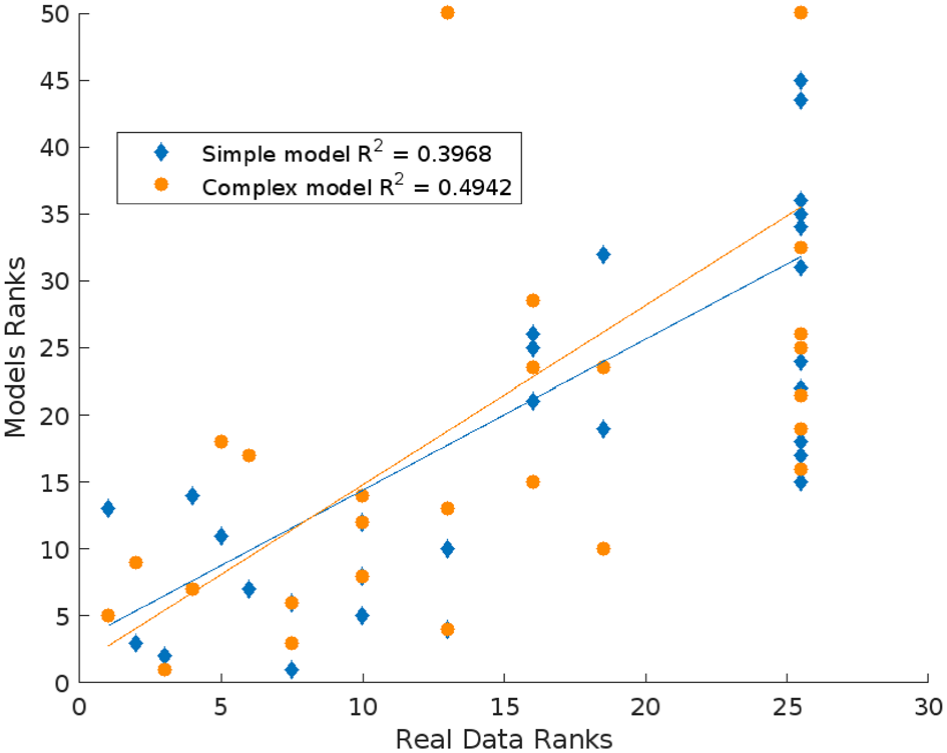
Scatterplot of the ranking of the appearing motifs in the real data and the motif ranking of the simple (blue diamonds) and complex (orange circles) model. The lines represent linear fits of the data.

Figure 3 shows the normalized distribution of the most common motifs in the aggregated networks of the real data and those of the two models. The frequency of motifs, especially in the networks of the real data and the complex model, shows a fast decay (Supplementary Fig. 1 for more details of motif frequencies). Thus, in these dolphins’ social networks only a few specific motifs are common whereas the rest of possible motifs are scarce or absent from the networks. 70% of the 20 most common motifs of the real data were present in the simple model and 80% in the complex model. We obtained a strong positive correlation between the motif ranking of the real data and the one of the simple model (*r*_*s*_ = 0.73, p < 0.001), and also between the motif ranking of the real data and the one of the complex model (*r*_*s*_ = 0.76, p < 0.001). In turn, Fig. 4 shows the scatter plot of the ranking of the appearing motifs in the real data versus the motif ranking of both models. We obtained a better correlation coefficient for the motif ranking of the complex model (R^2^ = 0.49) than for the simple one (R^2^ = 0.39).

## Discussion

In this work, we applied some of the methods coming from the network approach to the study of social networks of two groups of captive dolphins. In this way, we could study the structure of affiliative and aggressive interactions in bottlenose dolphins and the presence and influence of post-conflict resolution strategies in this species.

In general, data on the association levels and the social networks of both groups of captive dolphins were consistent with the association patterns observed in the wild^46,47^ and a study with captive animals^48^. The IAs of the two groups indicated that affiliation was higher among members of the same sex compared to mixed-sex pairs. Furthermore, the IAs of G1 were higher among males than among females. These outcomes also matched the pattern of relationships observed in wild groups of dolphins in which the most-stable bonds are those between males^47^. The results of the analysis of the most common aggressive and affiliative motifs of the temporal networks were in line with the obtained indices of affiliation. Among the most common motifs in the temporal networks of both groups were the ones including female-female and male-male affiliative interactions in the same period.

We observed some differences between the social structure of the networks of G1 and G2. The main difference was that in G2 some male–female pairs presented a high IA. Furthermore, one of the most common motifs of the temporal networks of G2 was the one including a triangle of positive links between one of the females and the two males. This outcome coincides with the obtained IA for two of the male–female pairs of the group. These differences could be due to the reproductive state of one of the females of G2, Stella, which was sexually receptive during some of the days of the study (trainers’ personal communication). It has been shown that the relationships between male and female dolphins in the wild are short and unstable in time and are influenced by the different reproductive strategies of males and females^47^. Furthermore, there are several reports of wild male dolphins with high levels of association with sexually receptive females^48–50^. Therefore, the elevated indexes of affiliation between this female and the two adult males could reflect the reproductive state of Stella and the interest of the males in her. Nevertheless, although these results go in line with results of previous studies, we want to emphasize that we didn’t use any of the sex related differences in behavior for the modeling due to the small sample size of our study in terms of animals. We believe that generalizing the behaviors to males and females wouldn’t be supported by the statistics provided by the data.

The analysis of the temporal networks also indicated that, in both groups, the periods in which an affiliative contact took place were more numerous than the ones in which an aggressive contact occurred. Moreover, the time-aggregated networks revealed that affiliative contacts between dolphins often lasted several minutes whereas aggressive interactions were usually short, as reflected in the mean weight of the links of these networks. These results match the findings of other studies reporting the rate and duration of affiliative and aggressive interactions in dolphins. For example, Harvey and collaborators^48^ observed that in another group of captive bottlenose dolphins the most common social behaviors were affiliative interactions among them. Furthermore, several studies have reported that, in general, dolphins present low rates of agonistic behavior^3,38,48^. In addition, we found that, in both groups, the highest rate of aggression corresponded to male–female pairs. This result is in line with previous studies reporting a high rate of aggression between mixed-sex pairs^48^. It has been suggested that this high rate of agonistic behavior between male–female dyads could be due to the sexual coercion of males over females^51^. Given that, in both groups, females were the main receivers of the attacks from male dolphins the hypothesis of sexual coercion seems to apply to this case.

We built two models of affiliative and aggressive interactions to examine the dynamics of social behaviors in a small dolphin group. With these models, we also aimed to assess the presence and influence of post-conflict behaviors (reconciliation, new affiliations, and aggressions) on the structure of dolphins’ social networks. In these two models, we did not consider the sex or the identity of the dolphins.

The results of the simple model already captured some of the dynamics observed in G1. Many of the affiliative and aggressive motifs obtained with this model were also present in the networks of the real data. In addition, there was a strong positive correlation between the motif ranking of this model and that of the real data, as reflected by the value of the r_s_. In turn, the addition of the pattern of conflict resolution previously observed in the complex model slightly improved the results of the simple model. The outcomes of the complex model predicted better than the simple one the dynamics and structure of the networks of the real data. Regarding the affiliative and aggressive motifs, 80% of the most common motifs of the real data were also present in the networks of the complex model. Furthermore, we also obtained a strong positive correlation between these two rankings of motifs. Therefore, these results suggest the presence and influence of active conflict resolution in this dolphin group.

Overall, the results of the models point to the importance of post-conflict strategies to solve conflicts in groups of dolphins. As reported by other studies^40,41^, bottlenose dolphins seem to display different post-conflict strategies to alleviate distress and reduce the costs of aggressive interactions. The outcomes of the models are quite good if we consider that they did not contain any information on sex, index of affiliation or the reproductive state of the animals. That is, the complex model was able to capture the observed dynamics of this group of dolphins even though we only considered the general probabilities of post-conflict contacts. The small sample size has been a limiting factor for extracting solid conclusions on the influence of sex, affiliation index or reproductive state on the social structure of captive bottlenose dolphins. Therefore, future research with a larger sample size should try to address the influence of these factors on dolphins’ sociality, and study more complex motifs like triangles^19^.

Therefore, it is possible to add multiple variables and factors to these types of models to assess their influence on the social patterns of animal groups. The use of these models, thus, could be very useful to manage animal populations in captivity. For example, they could serve to calculate the optimum group size to avoid excessive conflicts in small spaces, improving animal welfare. Furthermore, monitoring the temporal networks of the population during different periods could shed light on relevant changes in group behavior and social interactions throughout the day or year. In this way, if the network analysis reveals an increase of aggressive contacts during certain seasons (e.g. females in oestrus), animal caretakers could anticipate this event and separate some of the individuals from the group. Excessive levels of aggression could be a serious problem in captive settings; thus, a better understanding of these dynamics could serve to improve the management of captive animal populations. These tools could also help to predict or monitor the process of adaptation of a new member of a group.

In conclusion, the network approach reveals as a useful tool to apply to the study of conflicts and social dynamics in animal groups. The analysis of temporal and aggregated networks has provided accurate results on the structure and pattern of interactions of bottlenose dolphins, matching features previously observed in both wild and captive settings. Thus, this type of analysis is a powerful and realistic method to study animal social patterns. Furthermore, assessing the main features and structure of animal social networks using models that simulate the observed dynamics can expand our knowledge of the social life of many gregarious species. Finally, the outcomes of these models can have important applications in the management of animal groups in captivity.

## Supplementary Information

Supplementary Information 1.

Supplementary Information 2.

Supplementary Information 3.

## Data Availability

All data generated or analyzed during this study are included in this published article (and its Supplementary Information files).
