# Supplementary material for: The study of aggression and affiliation motifs in bottlenose dolphins’ social networks
Source: Sci Rep. 2022 Nov 16;12:19672. doi: 10.1038/s41598-022-22071-w (PMC9668813; doi:10.1038/s41598-022-22071-w)
Supplement: Supplementary file 3 — Supplementary Information 3. [file 41598_2022_22071_MOESM3_ESM.docx]

**Supplementary information**

**Supp. Table 1** Probabilities of the different types of interactions used in the complex model

| **Probability** | **Definition** | **Formula** |
| --- | --- | --- |
| **Reconciliation per day (prd)** | Number of times that former opponents affiliated after each conflict divided by the total number of conflicts that day | $prd=\frac{\left( Numreconciliations \right)}{\left( Numconflictsperday \right)}$ |
| **Total probability of reconciliation (pr)** | All prd divided by the total number of days with conflicts | $pr=\frac{\sum prd}{\left( Numdayswithconflict \right)}$ |
| **New affiliations/aggressions per day (pfd/pgd)** | Number of times one of the opponents engaged in a new affiliative/ aggressive interaction with a third dolphin for each conflict, divided by the number of all possible affiliative/aggressive interactions that could have taken place after each conflict that day | ${pfd}/{pgd}=\frac{\begin{aligned} Num{aff}/{agg} \end{aligned}}{\begin{aligned} 4xnumconflictsthatday \end{aligned}}$ |
| **Total probability of new affiliations (pft) /aggressions (pgt)** | All pfd/pgd divided by the number of days in which a conflict took place | ${pft}/{pgt}=\frac{\sum{pfd}/{pgd}}{\begin{aligned} Numdayswithconflict \end{aligned}}$ |
| **Probability of spontaneous affiliation/aggression (psf/psg)** | Number of spontaneous affiliations/aggressions per day divided by the total number of periods of that day | ${psf}/{psg}=\frac{\begin{aligned} \left( Numspont{aff}/{agg} \right) \end{aligned}}{Numperiodsperday}$ |
| **Probability of spontaneous affiliation/aggression per period (psfp/psgp)** | All psf or psg divided by the number of recording days | ${psfp}/{psgp}=\frac{\sum{psf}/{psg}}{\begin{aligned} Numrecordingdays \end{aligned}}$ |

**Supp. Table 2** Number of affiliative and aggressive contacts of both groups

| **Pair** | **Number affiliative contacts** | | **Number aggressive contacts** | |
| --- | --- | --- | --- | --- |
|  | **Group 1** | **Group 2** | **Group 1** | **Group 2** |
| **M-F** | 318 | 105 | 571 | 73 |
| **M-M** | 501 | 292 | 30 | 22 |
| **F-F** | 486 | 111 | 37 | 5 |
| **Total** | 1305 | 508 | 638 | 100 |

**Supp. Fig. 1 a.** Frequency of the motifs of the aggregated networks of the real data, and mean frequency of the motifs of the time-aggregated networks **b**. of the simple, and **c.** complex model. The motif’s code reads as follows: No-interactions = 0, affiliative interactions = 1 and negative interactions = 2. Each digit position corresponds to a specific pair of dolphins (digit position: pair): 1: 1-2, 2: 1-3, 3: 1-4, 4: 2-3, 5: 2-4, 6: 3-4.


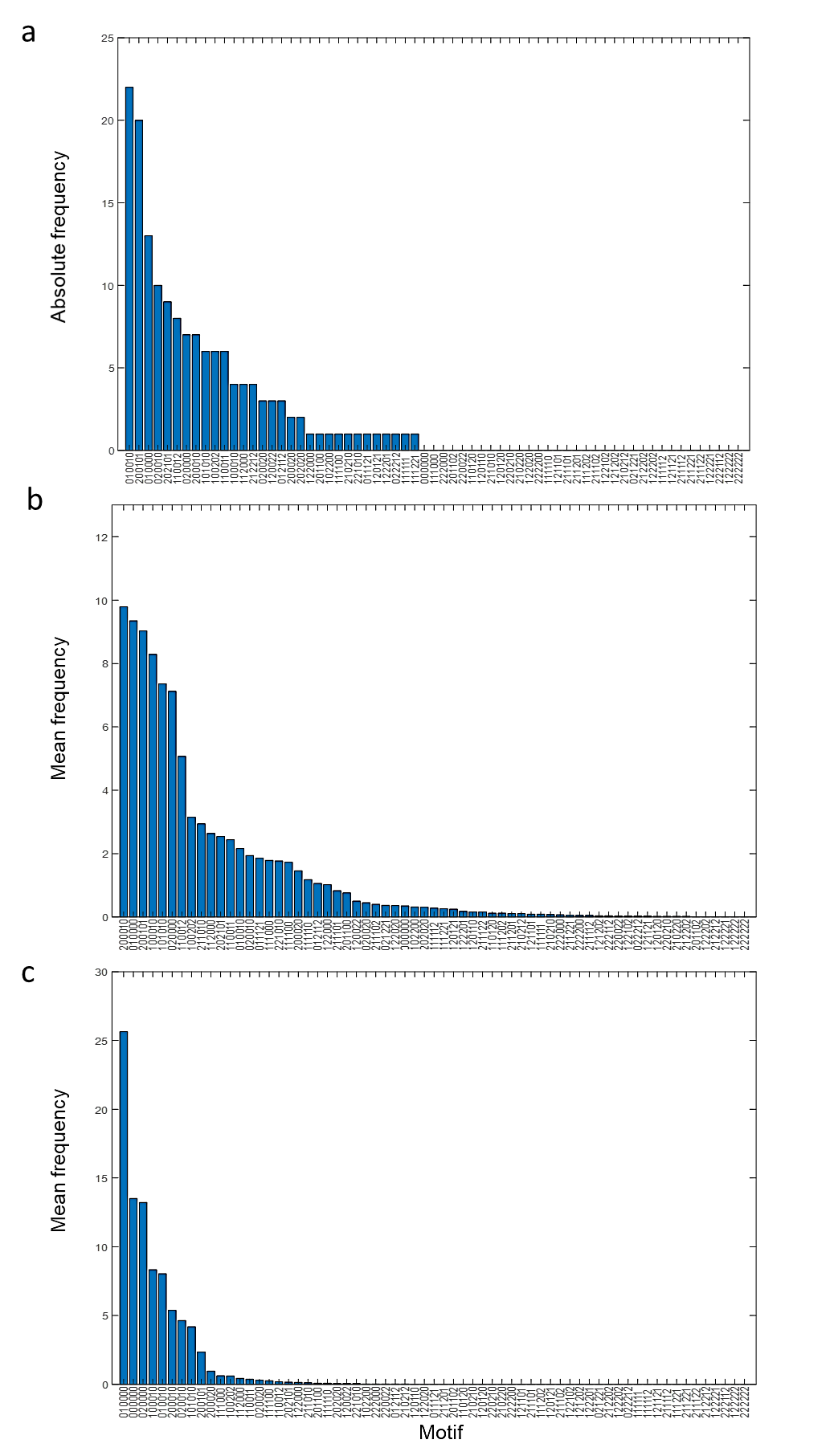


**Supp. Fig. 2** Temporal networks of G1 for each day. P is the number of the period, red links represent aggressive interactions and blue links affiliative interactions.


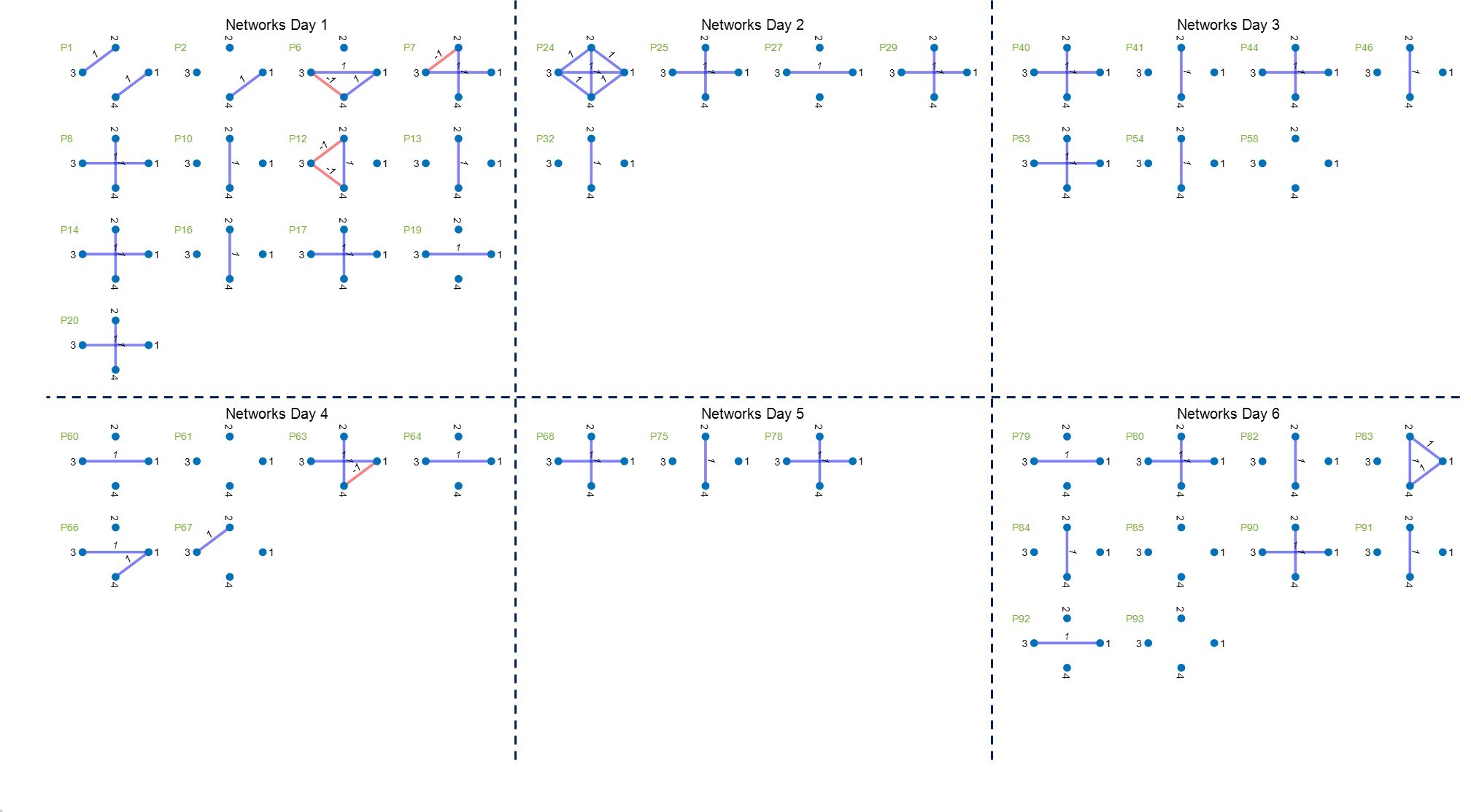


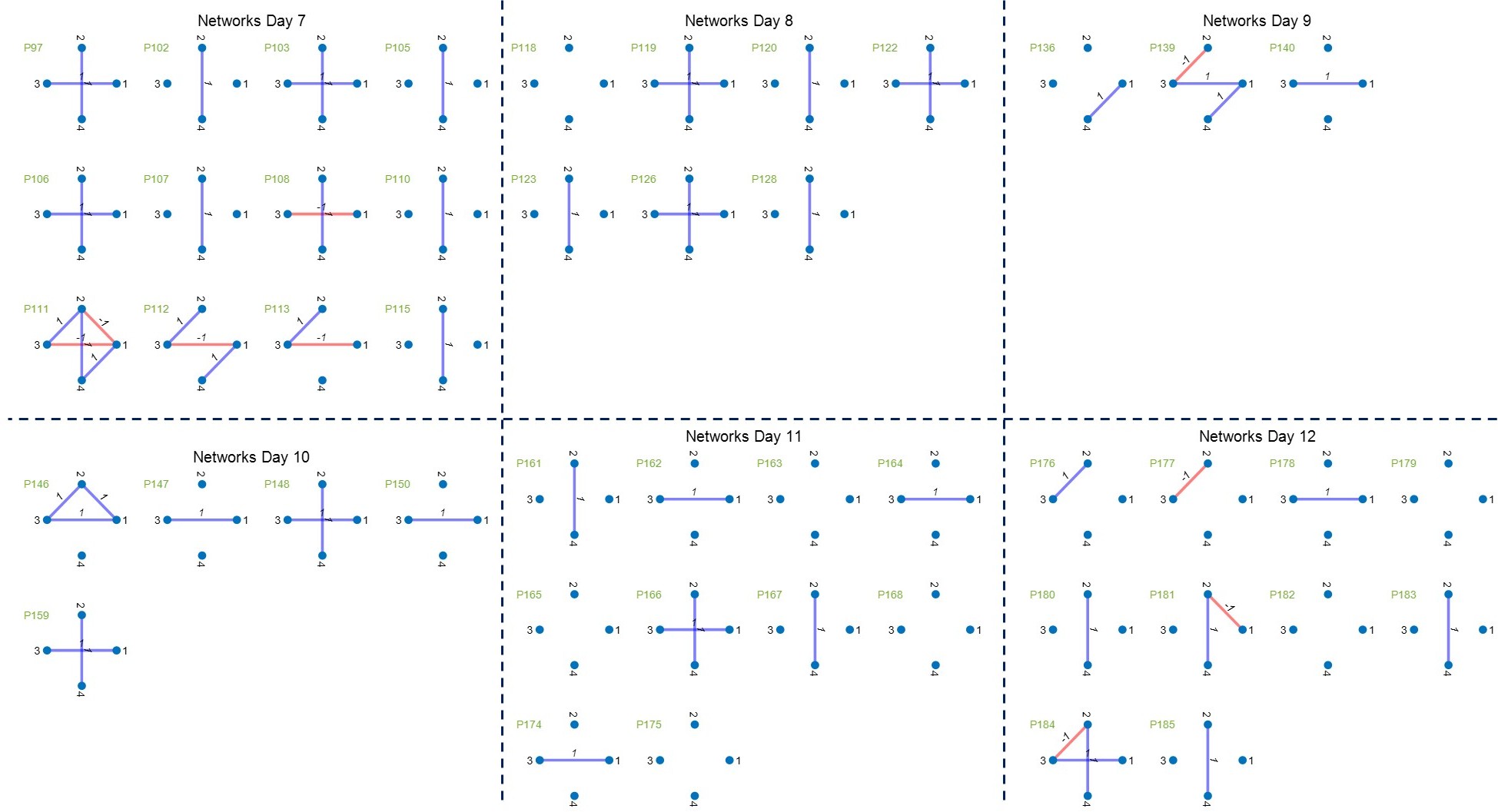


**
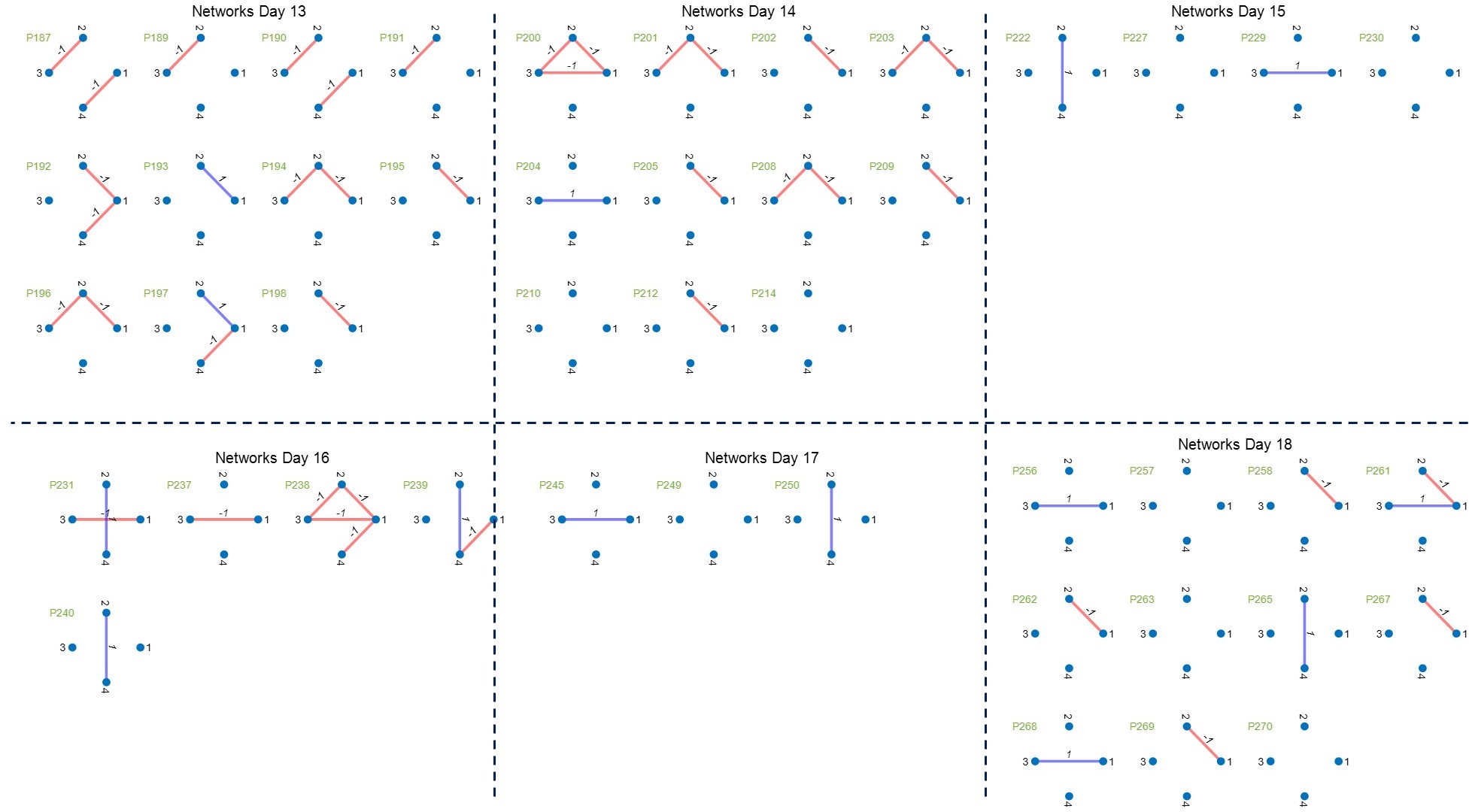
**


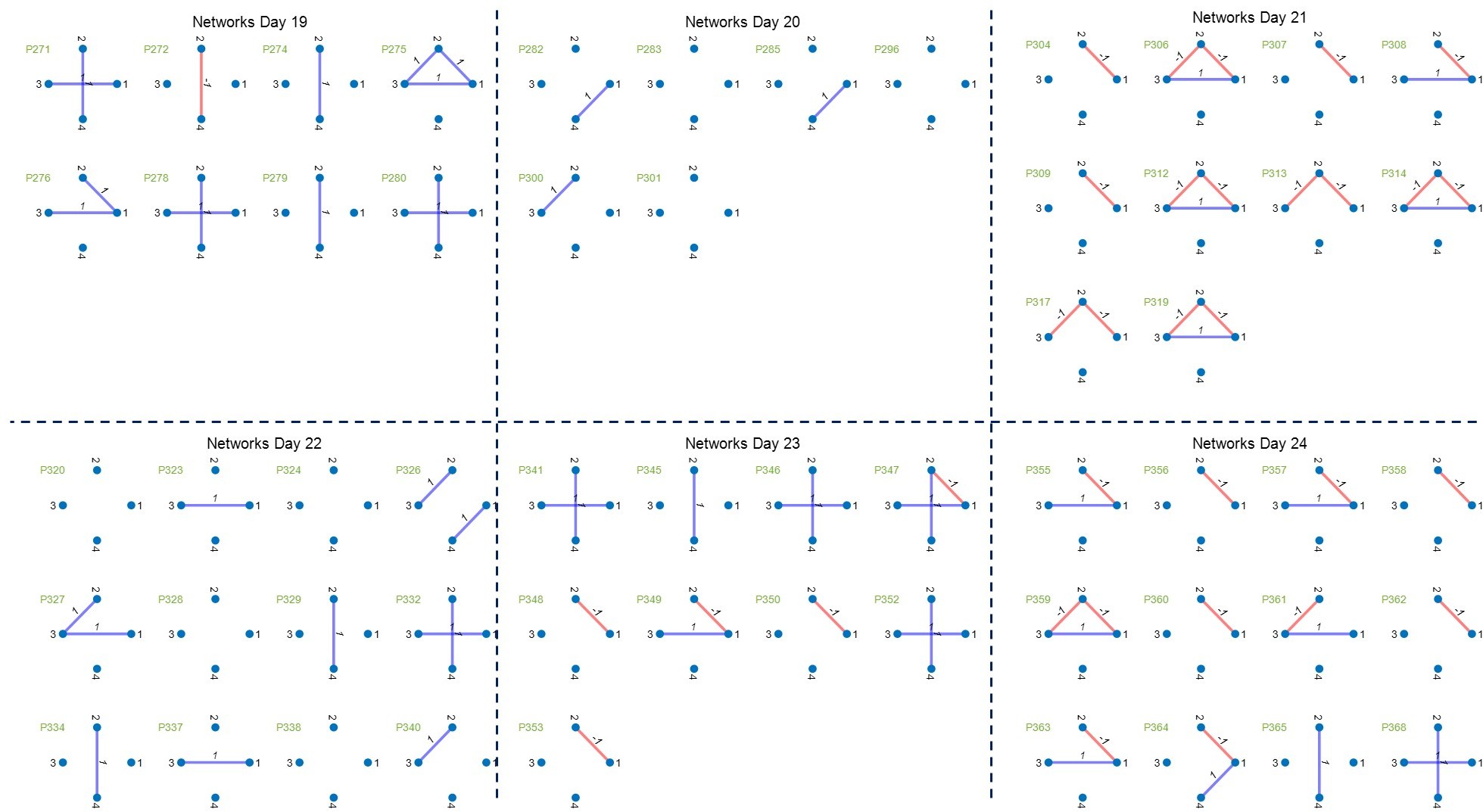


**
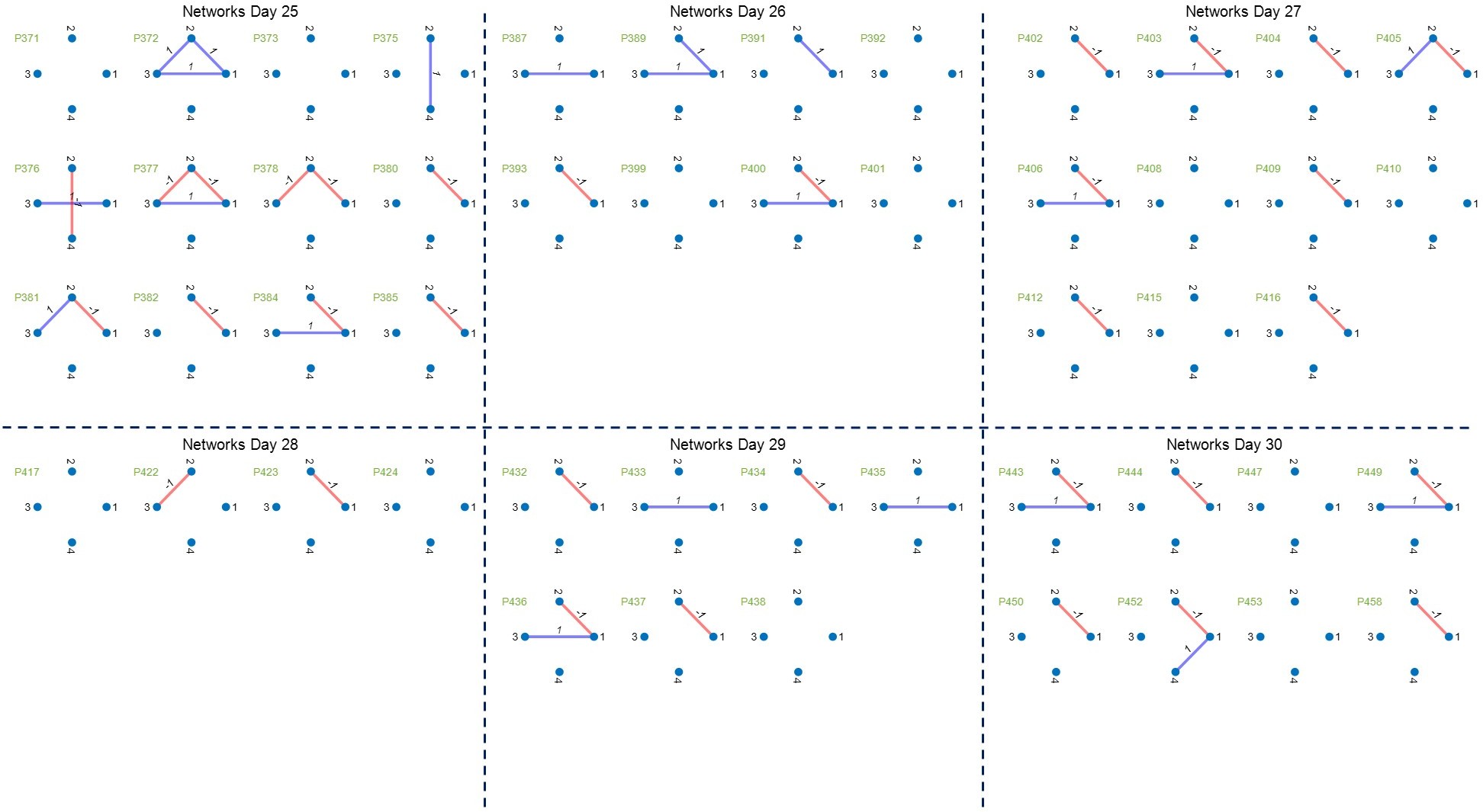
**


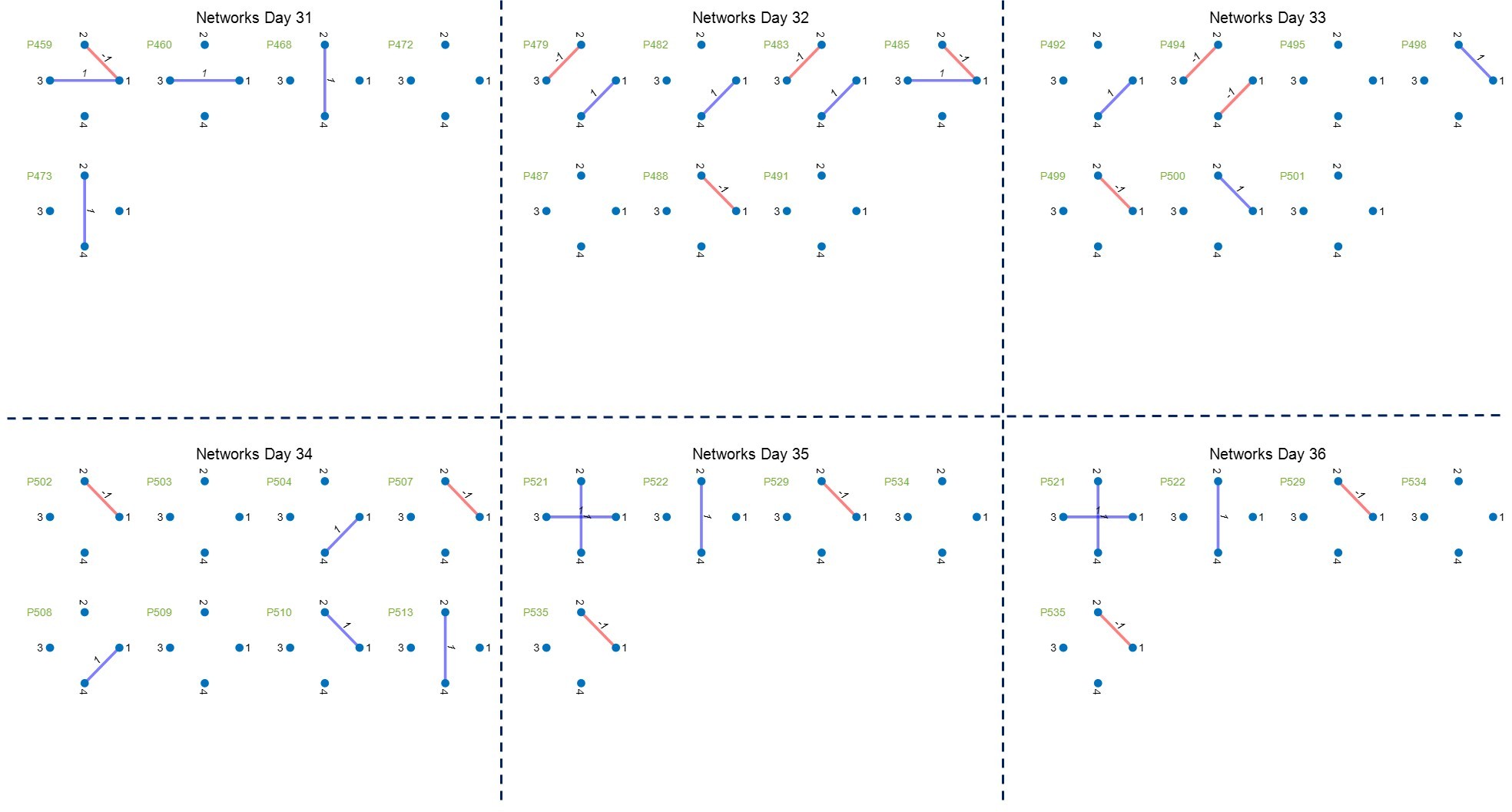


**
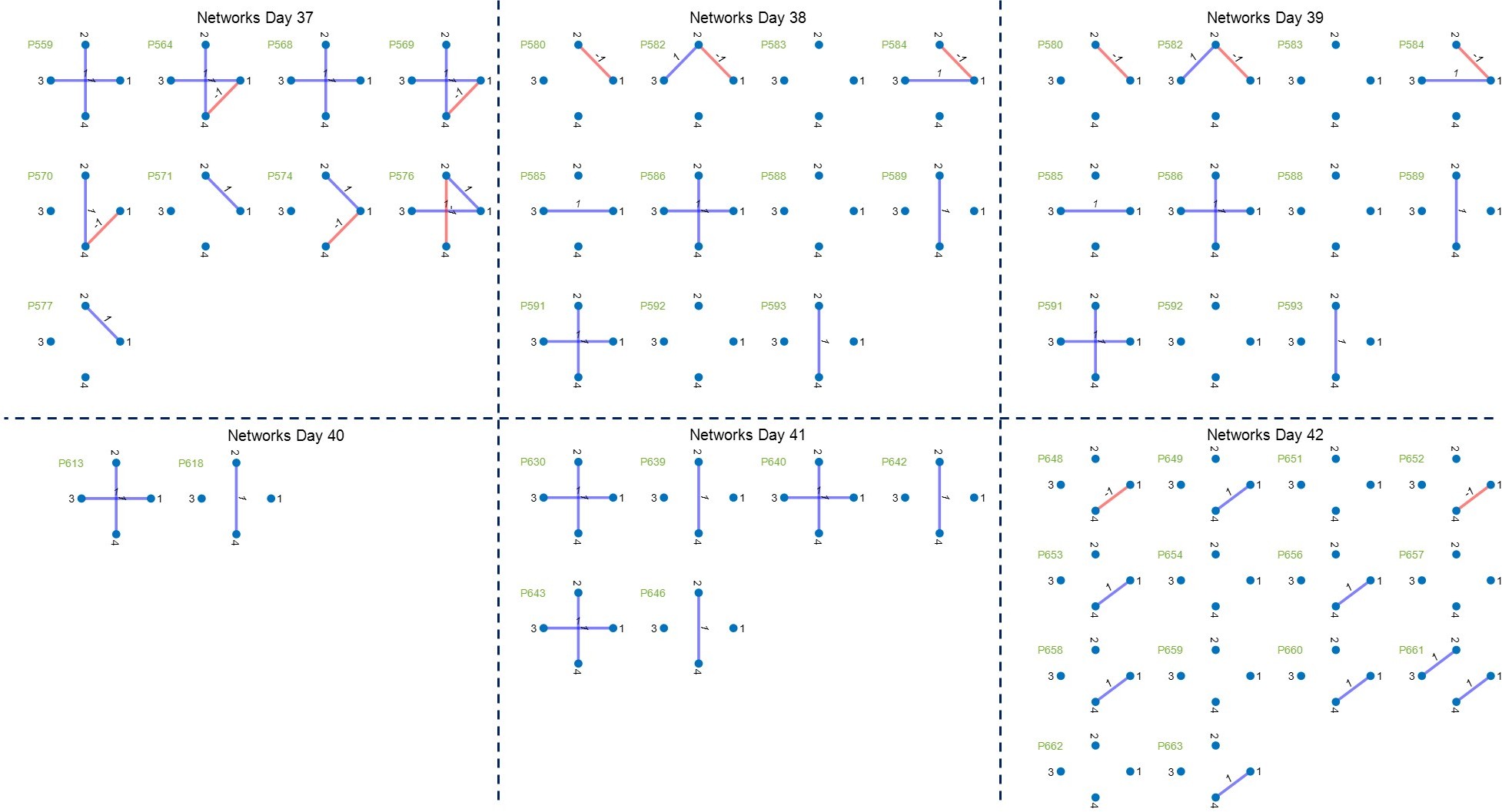
**


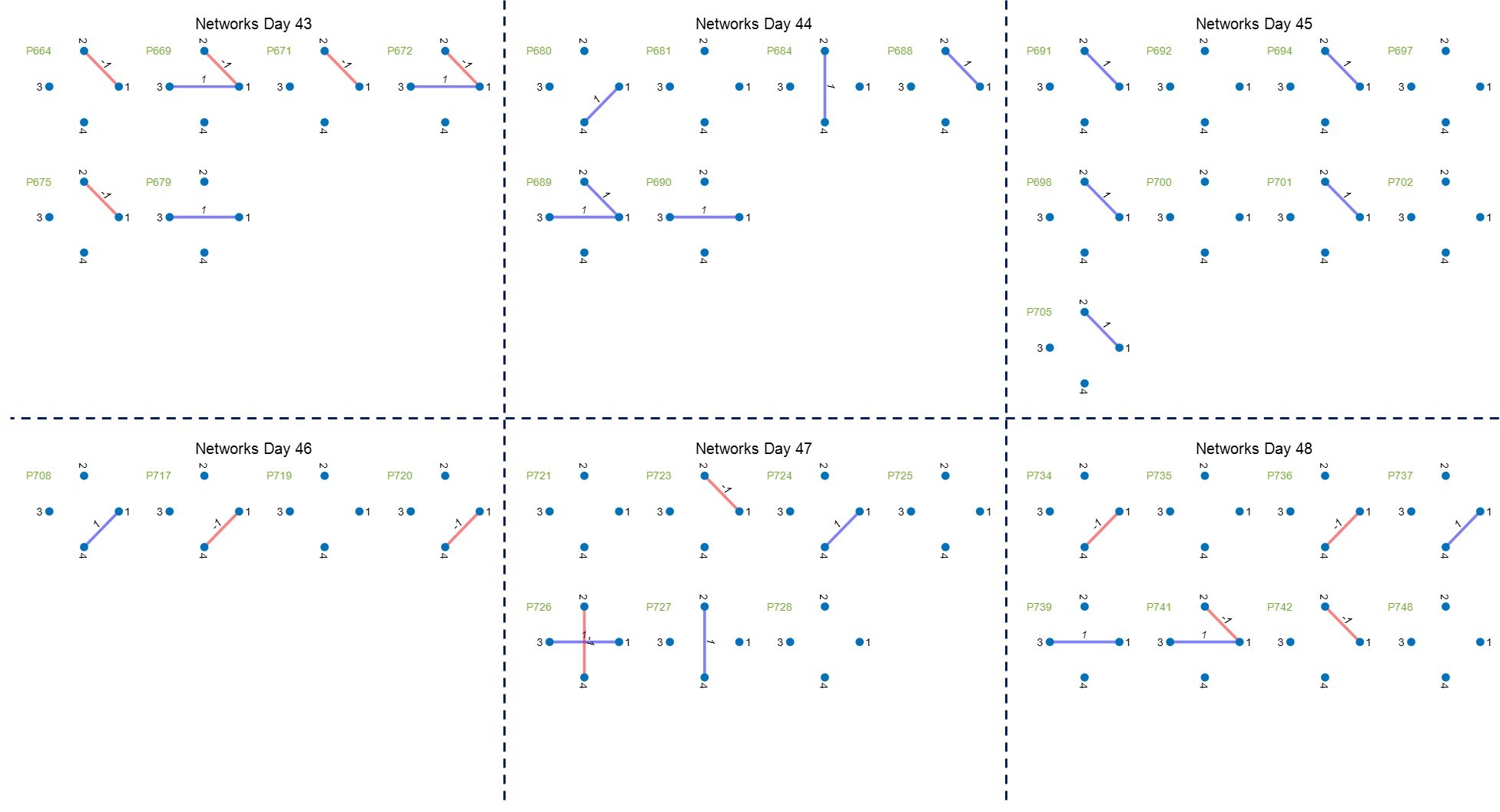


**
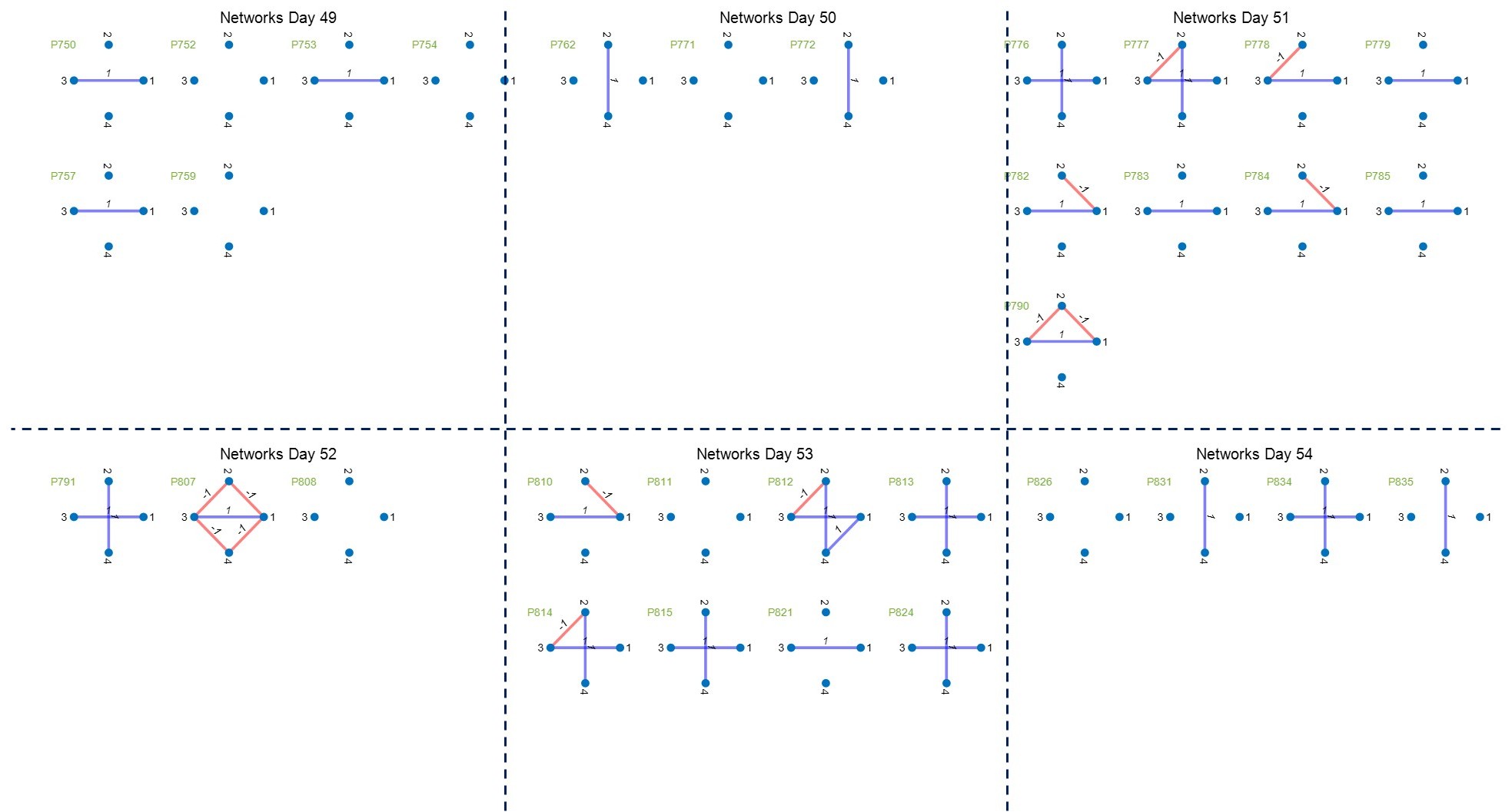
**


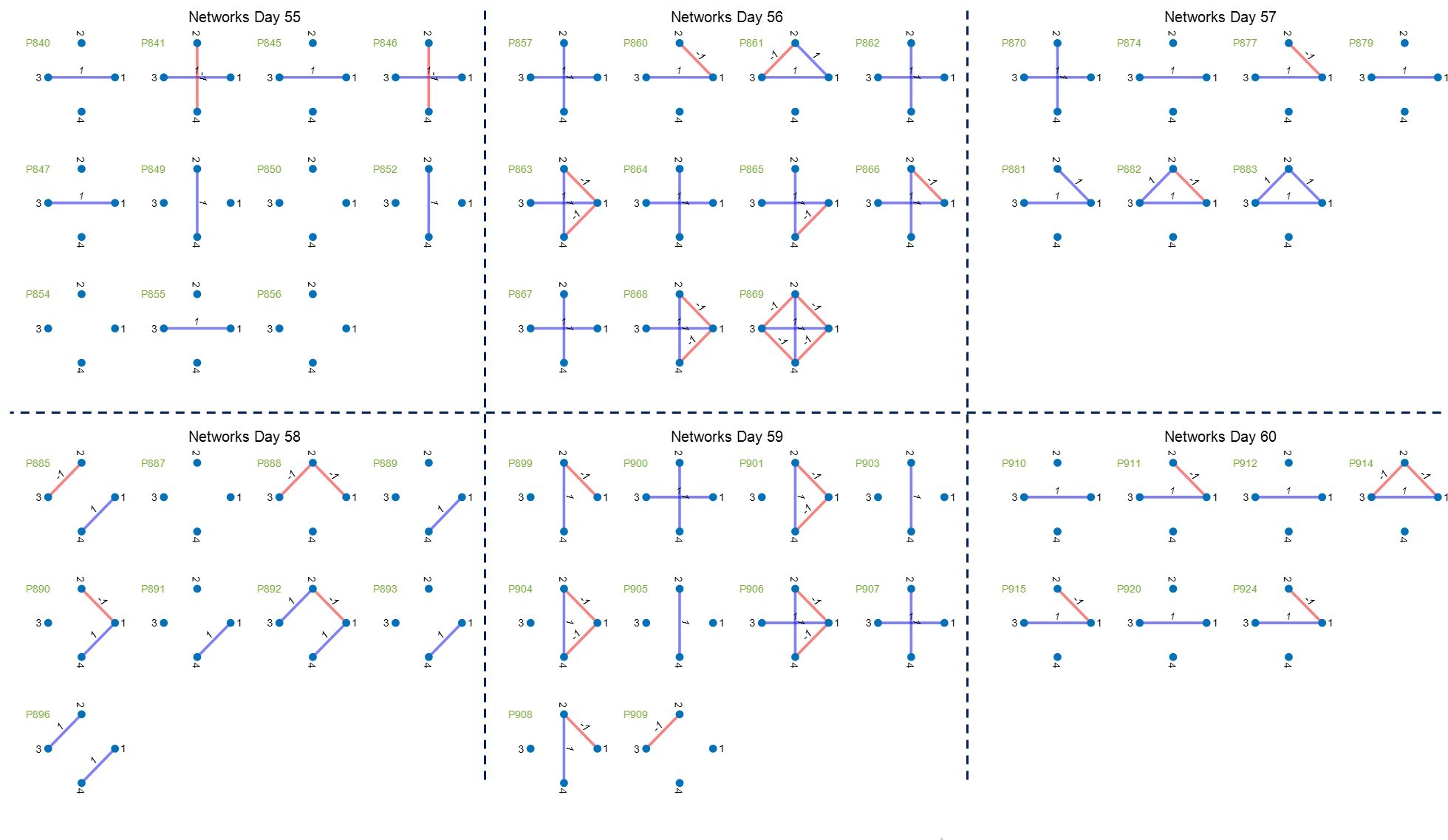


**
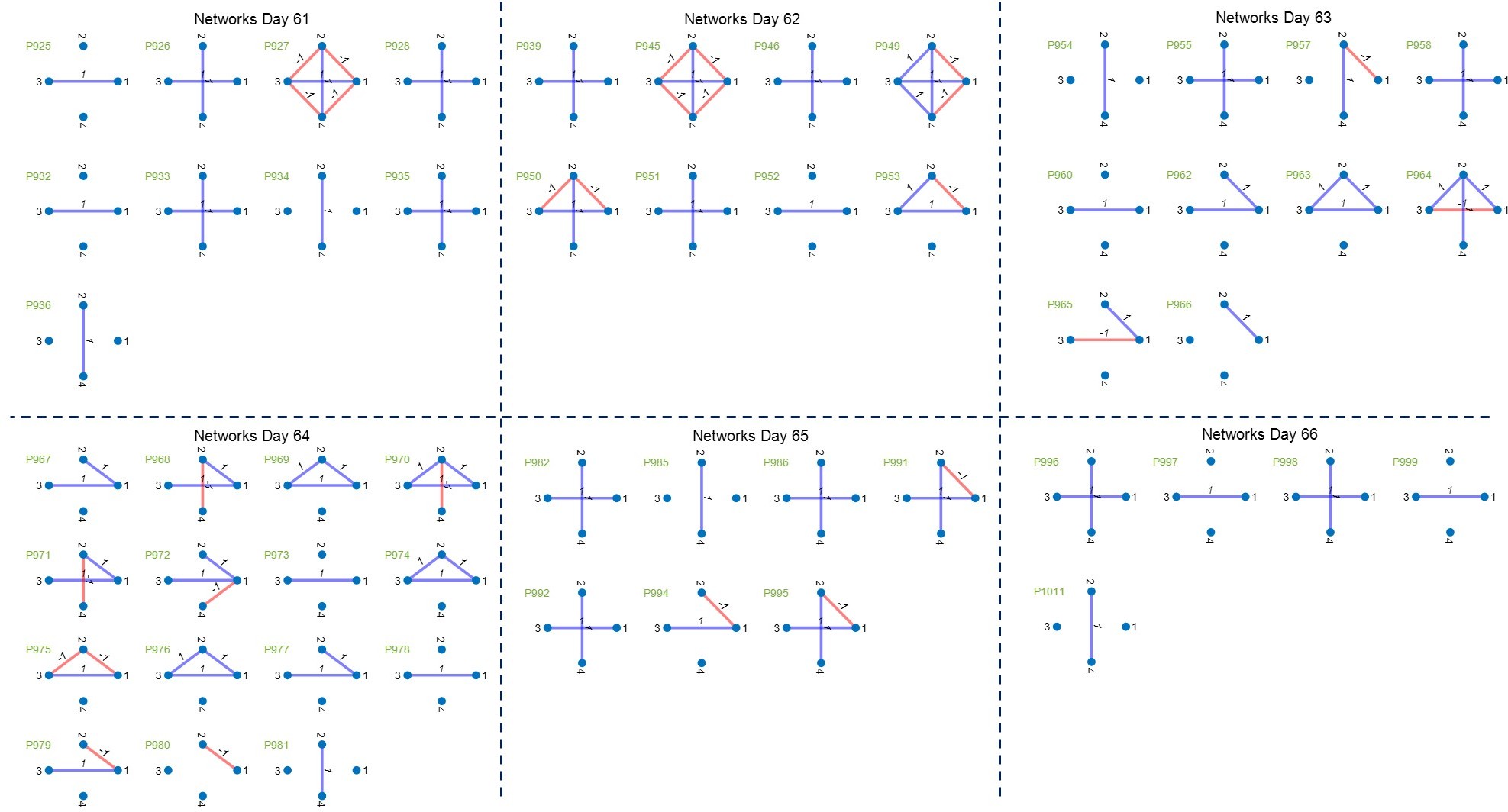
**


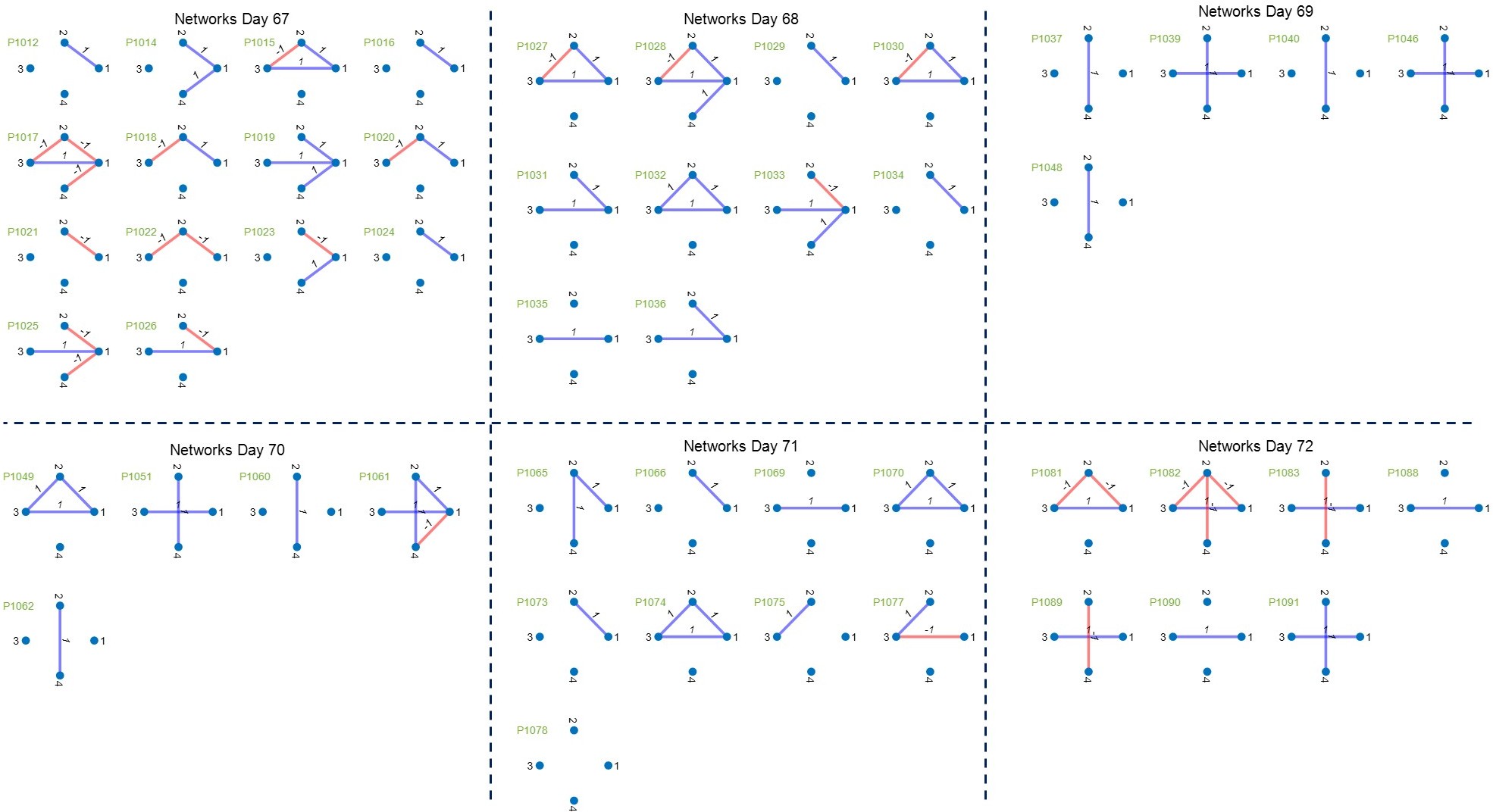


**
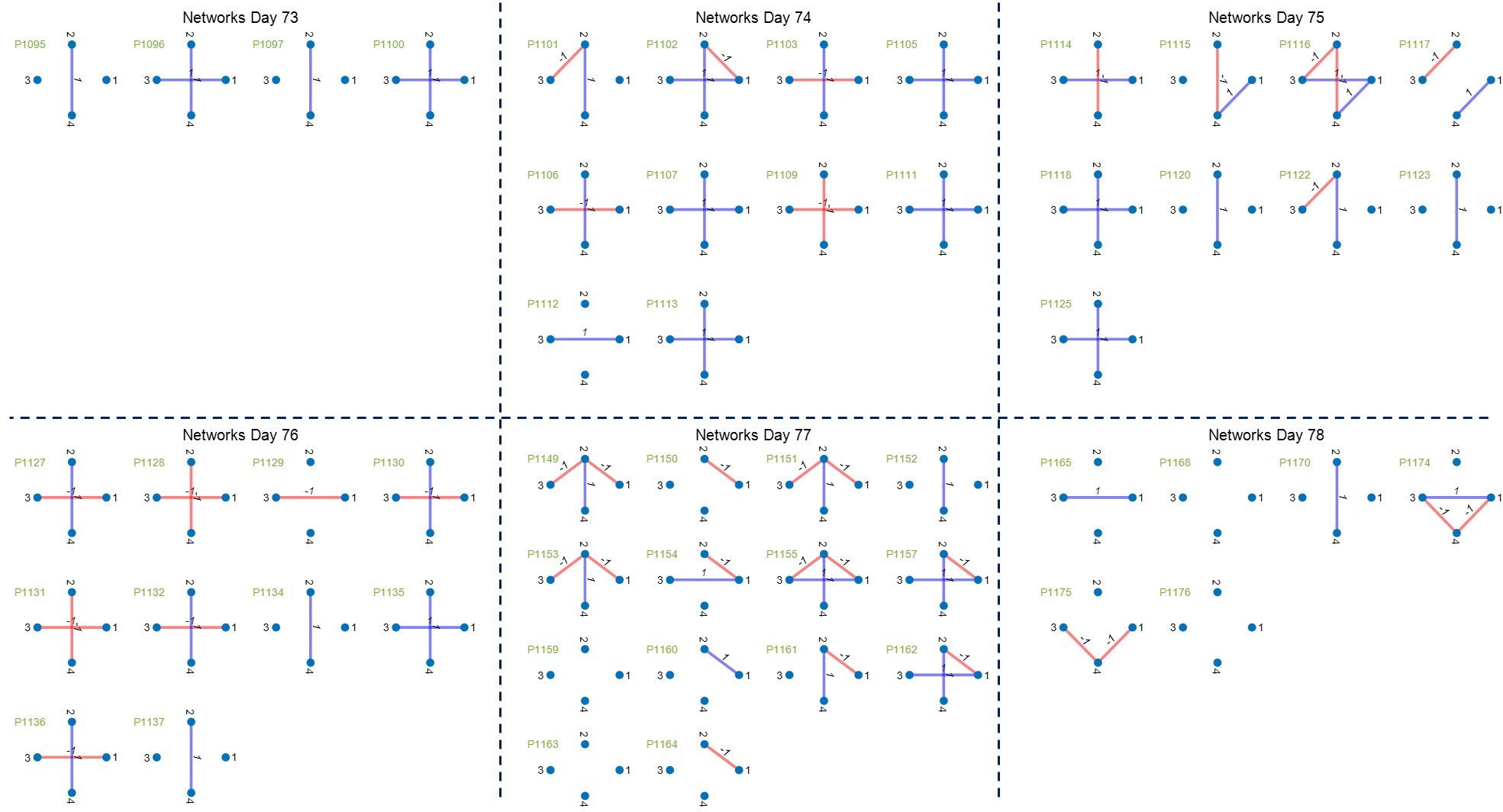
**


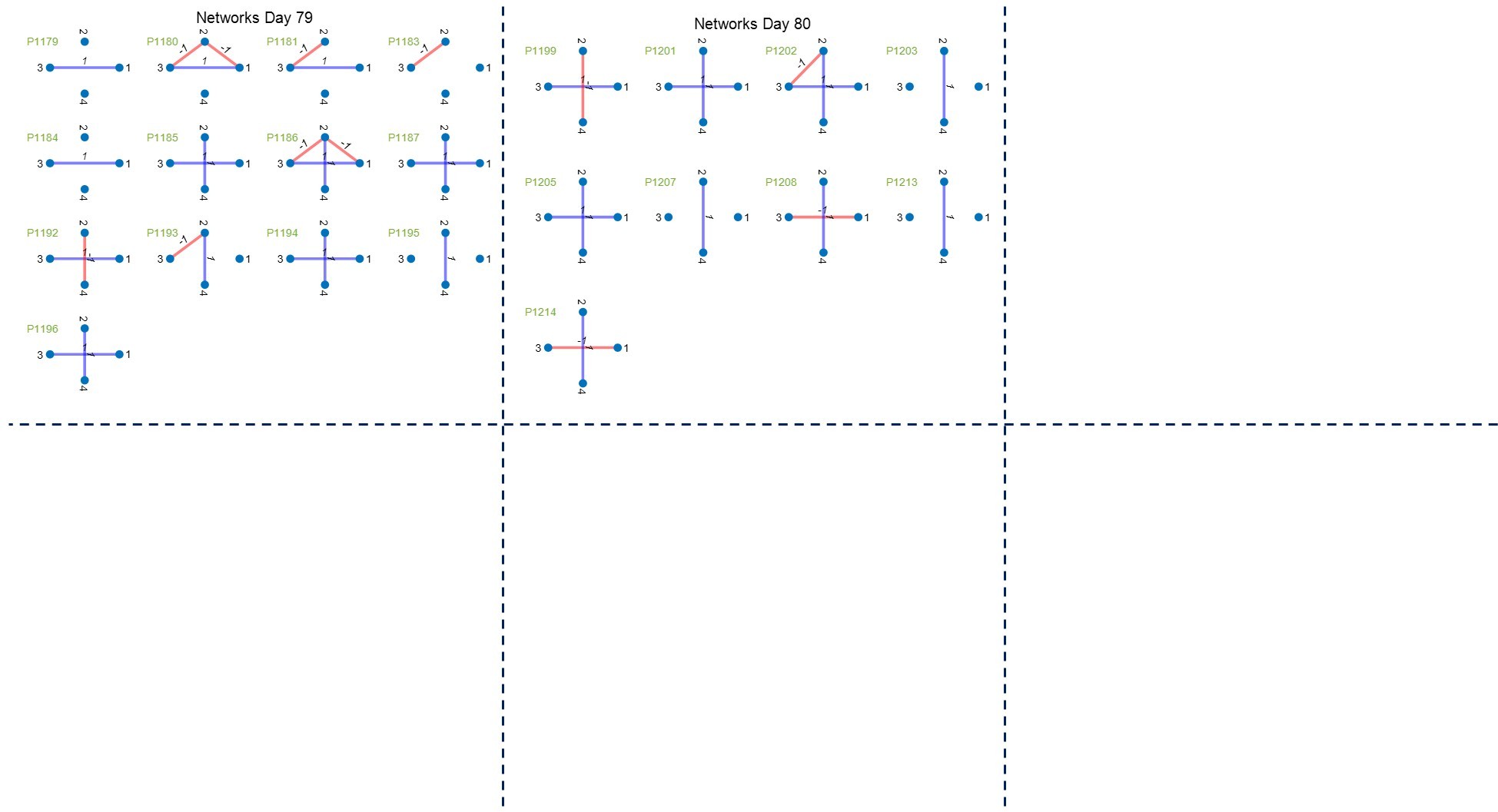


**Supp. Fig 3** Time aggregated networks of G1. The number accompanying the links indicate their weight. Red links represent aggressive interactions, blue links affiliative interactions.

**
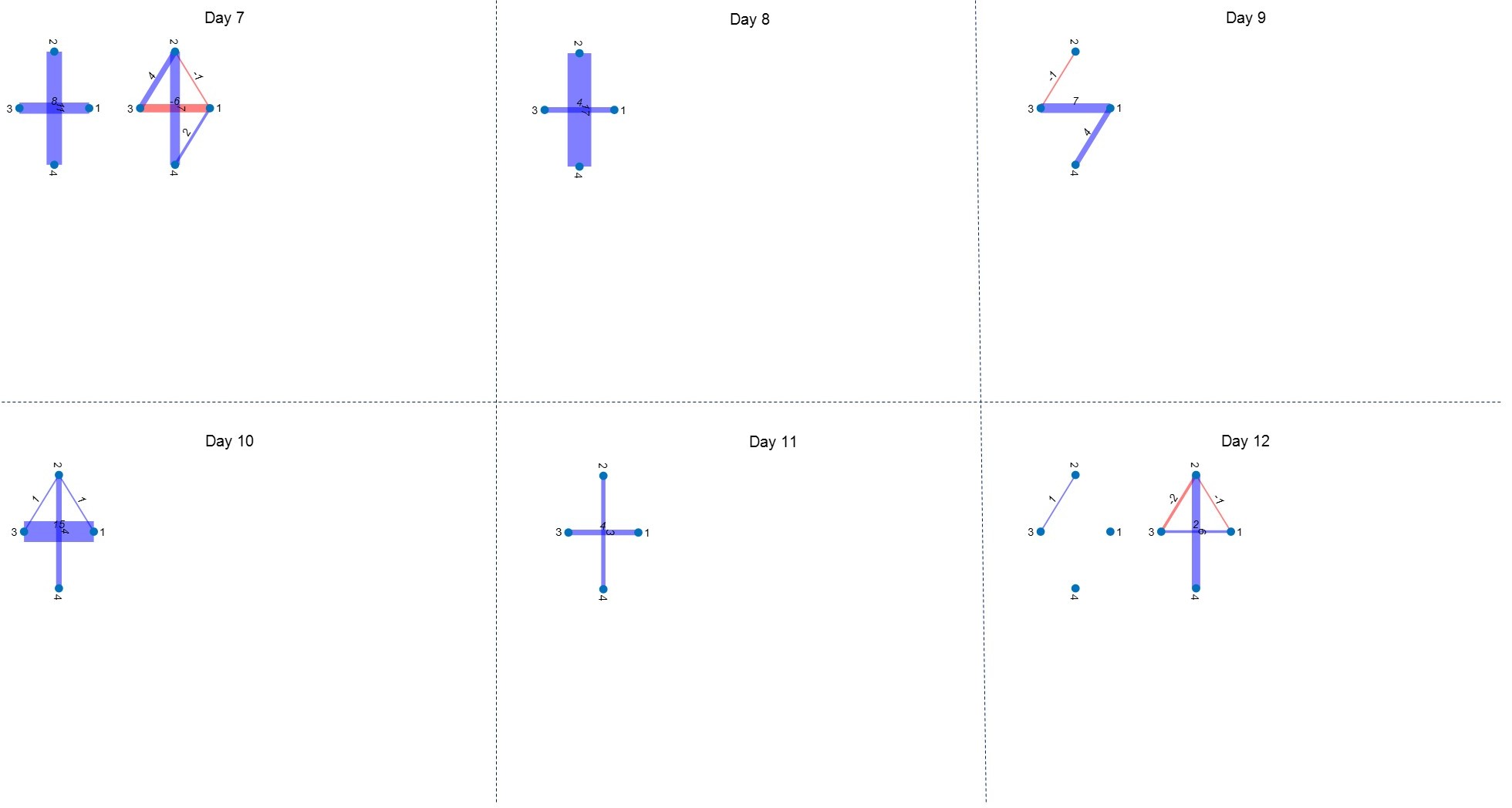
**


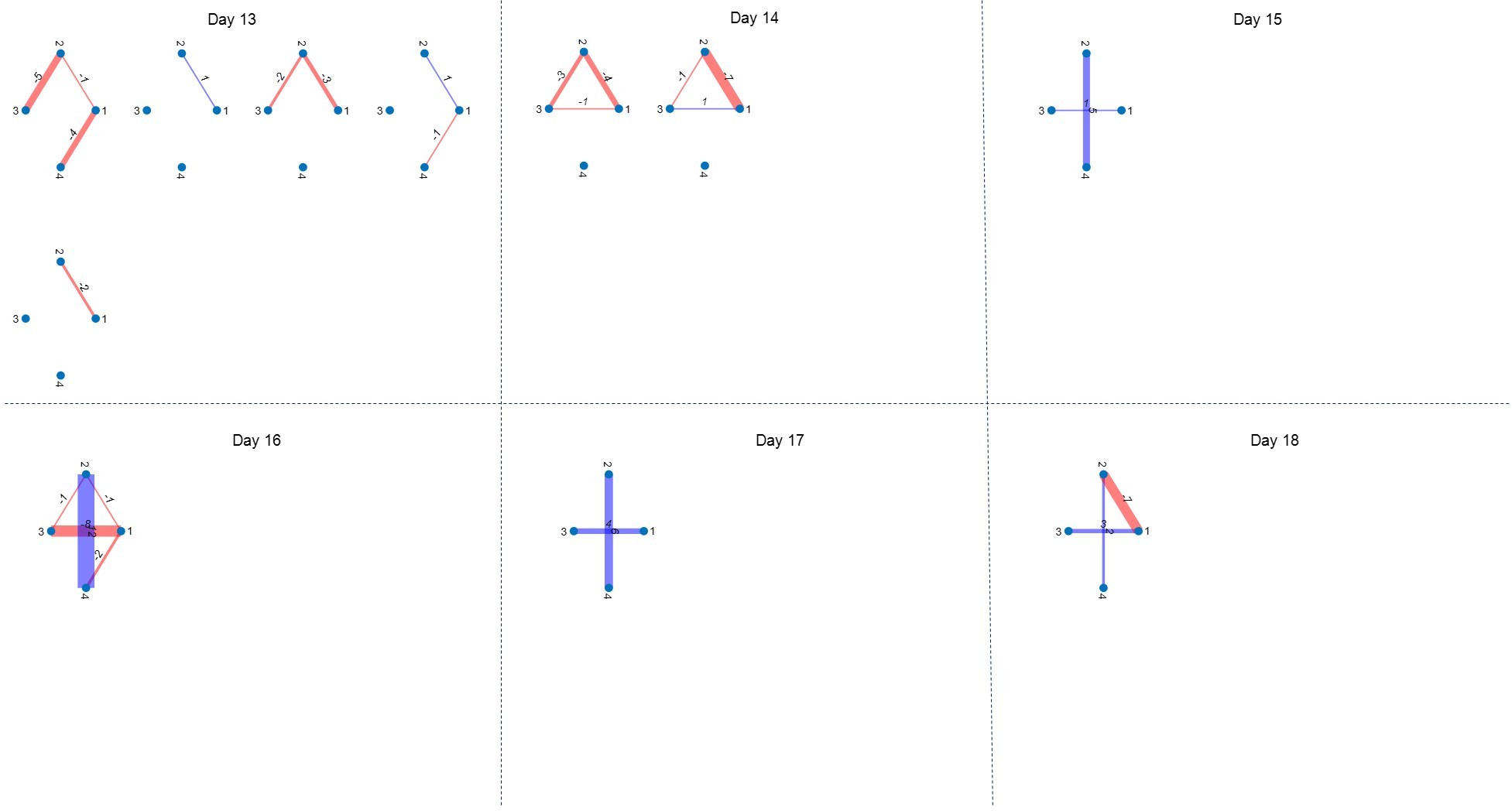


**
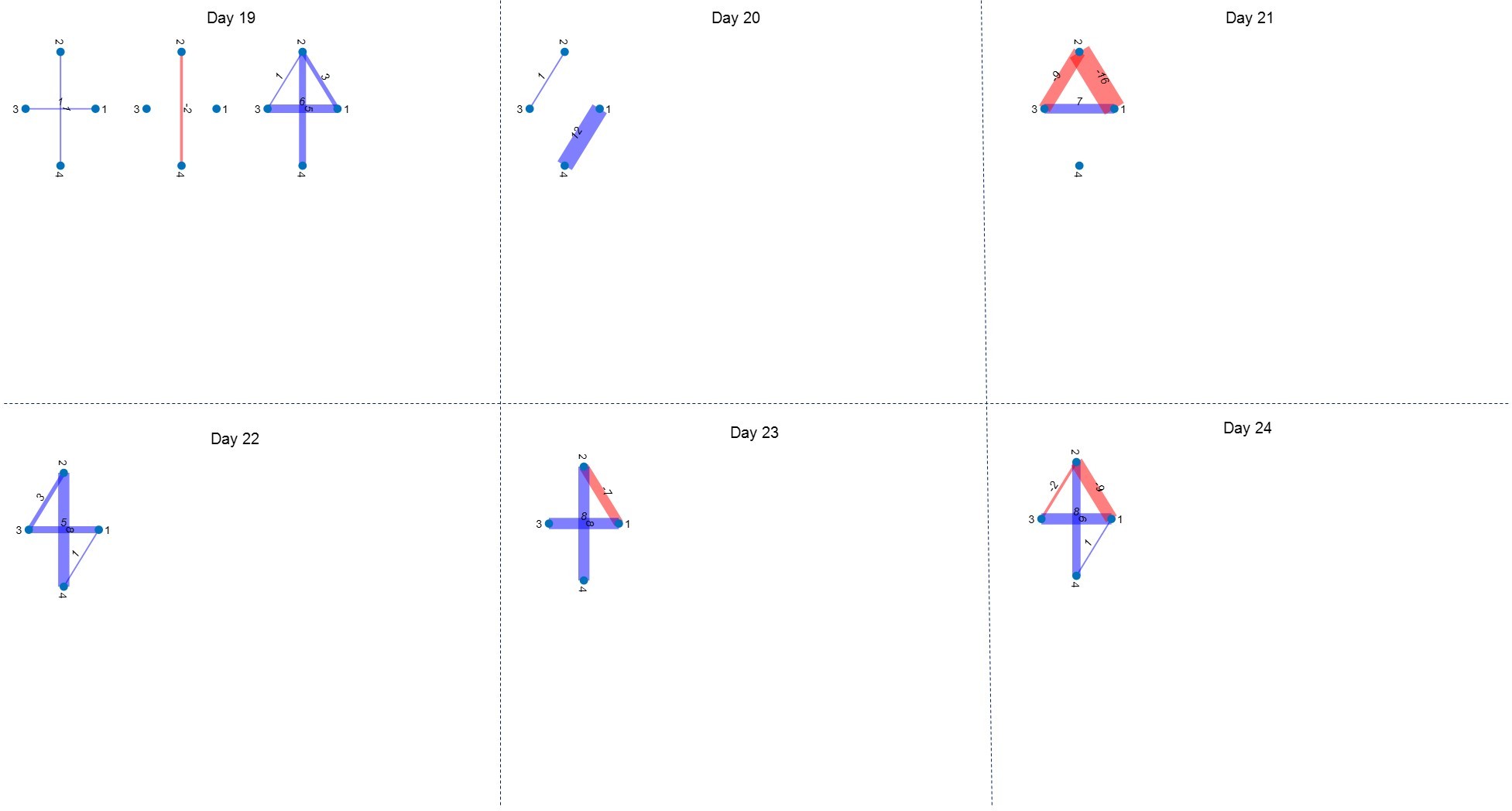
**


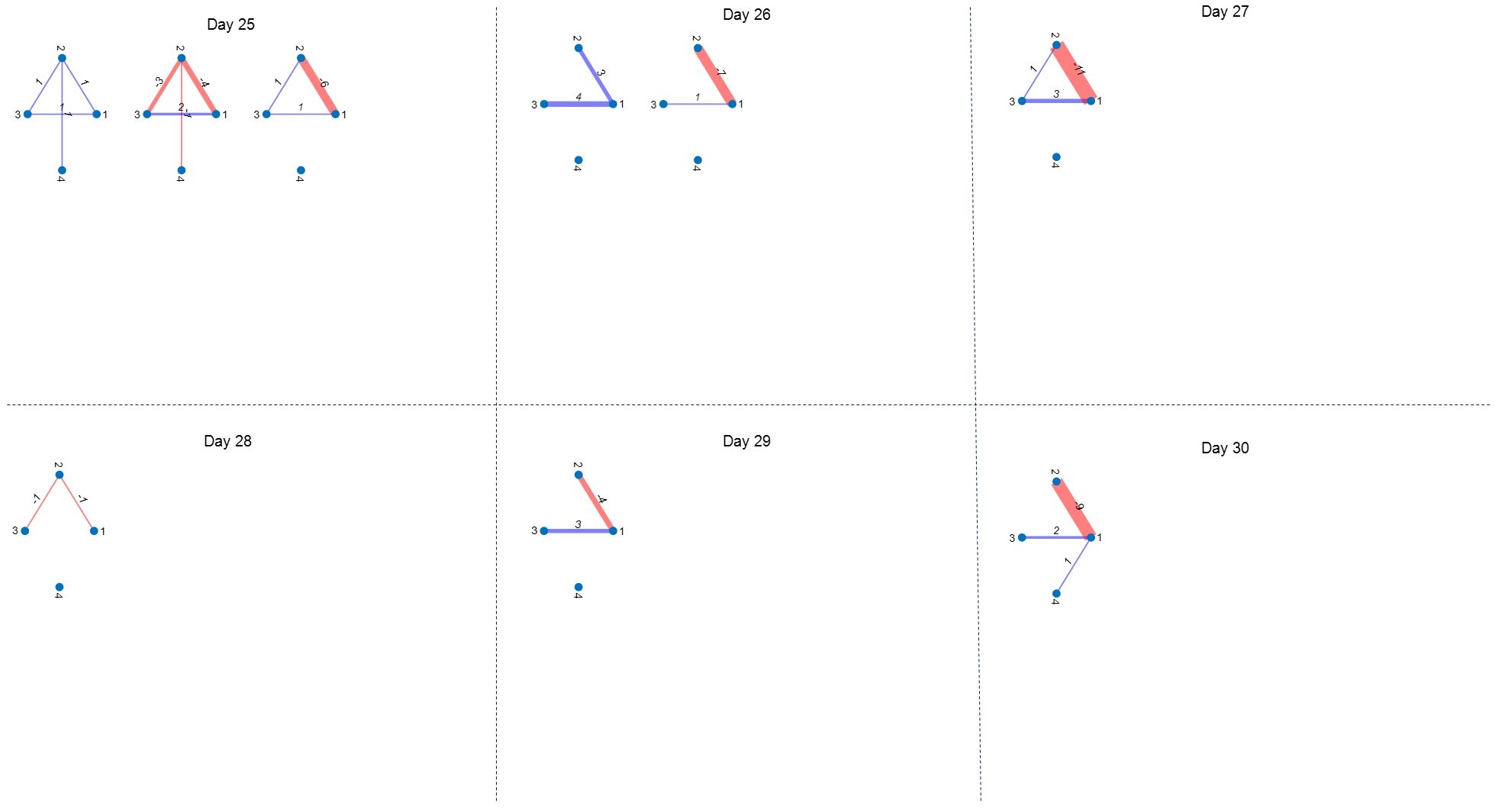


**
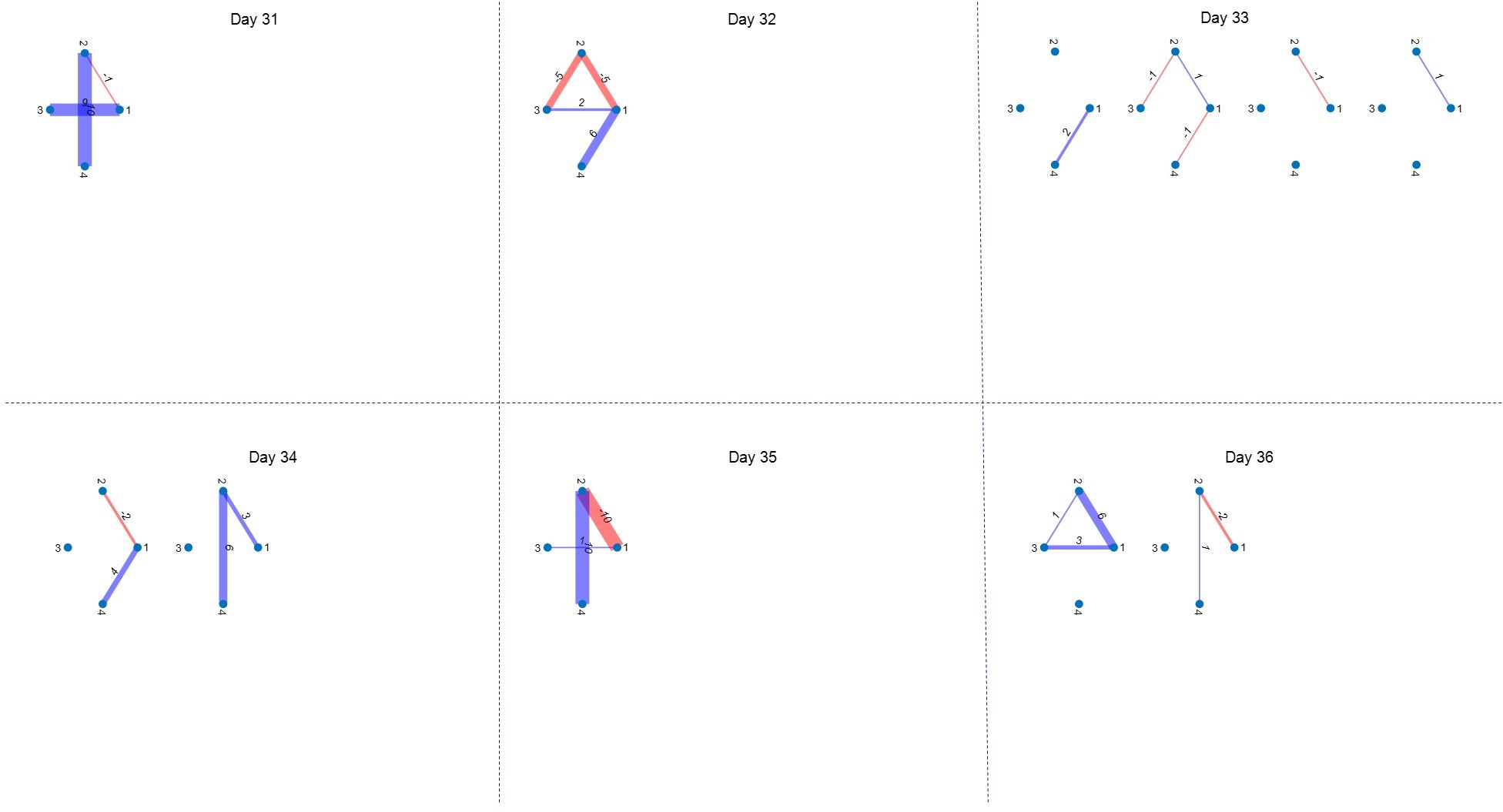
**


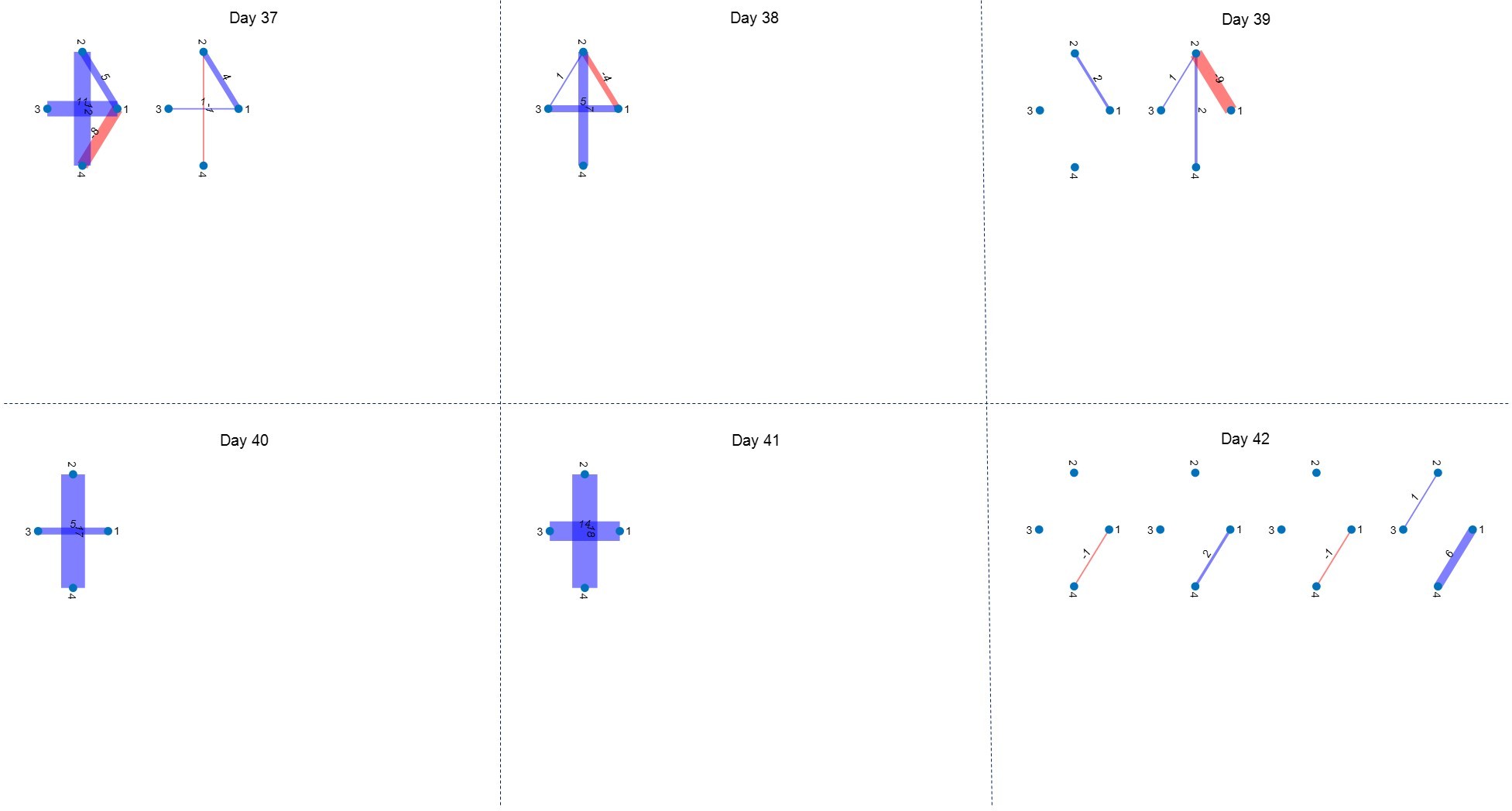


**
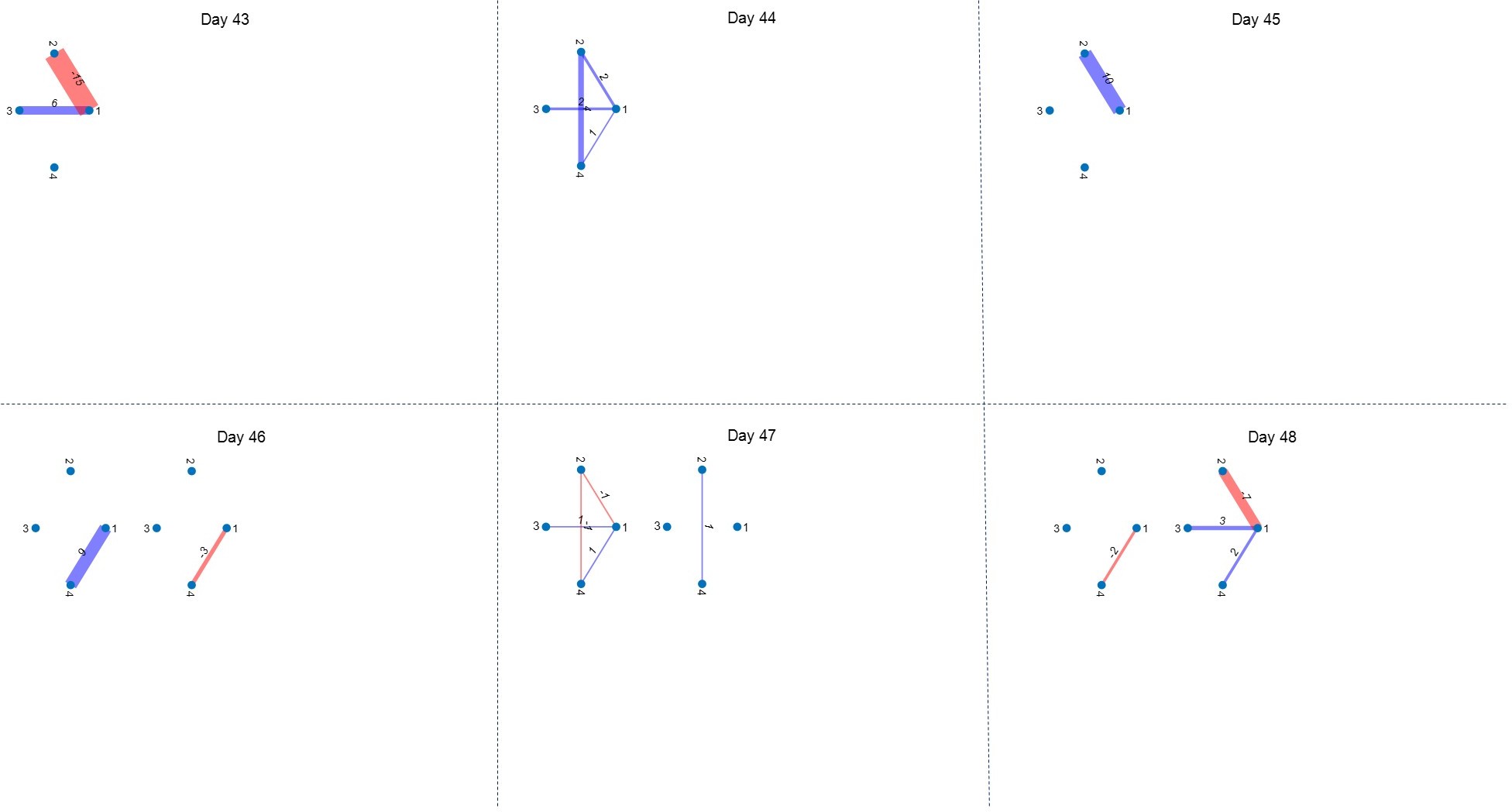
**


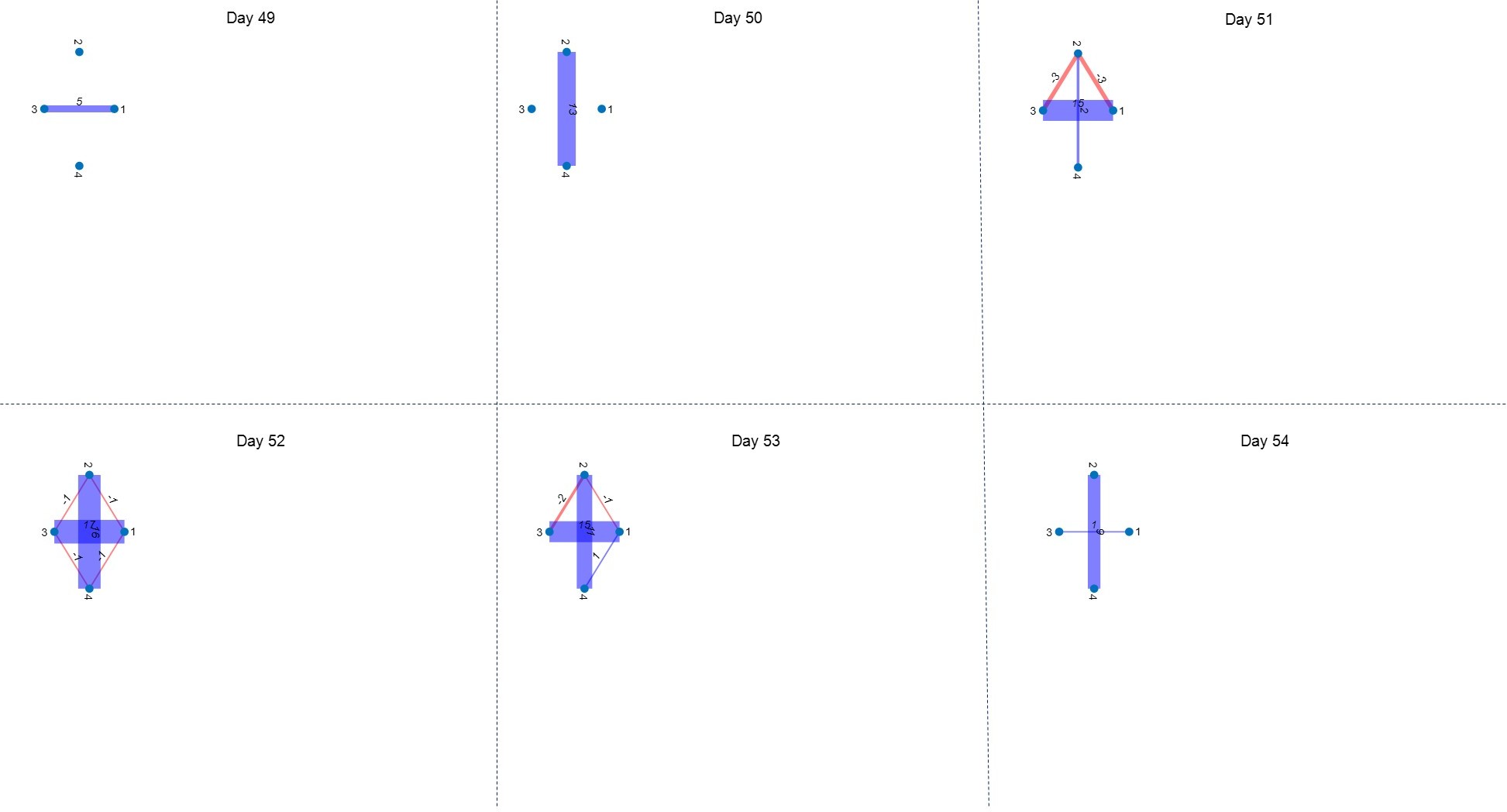


**
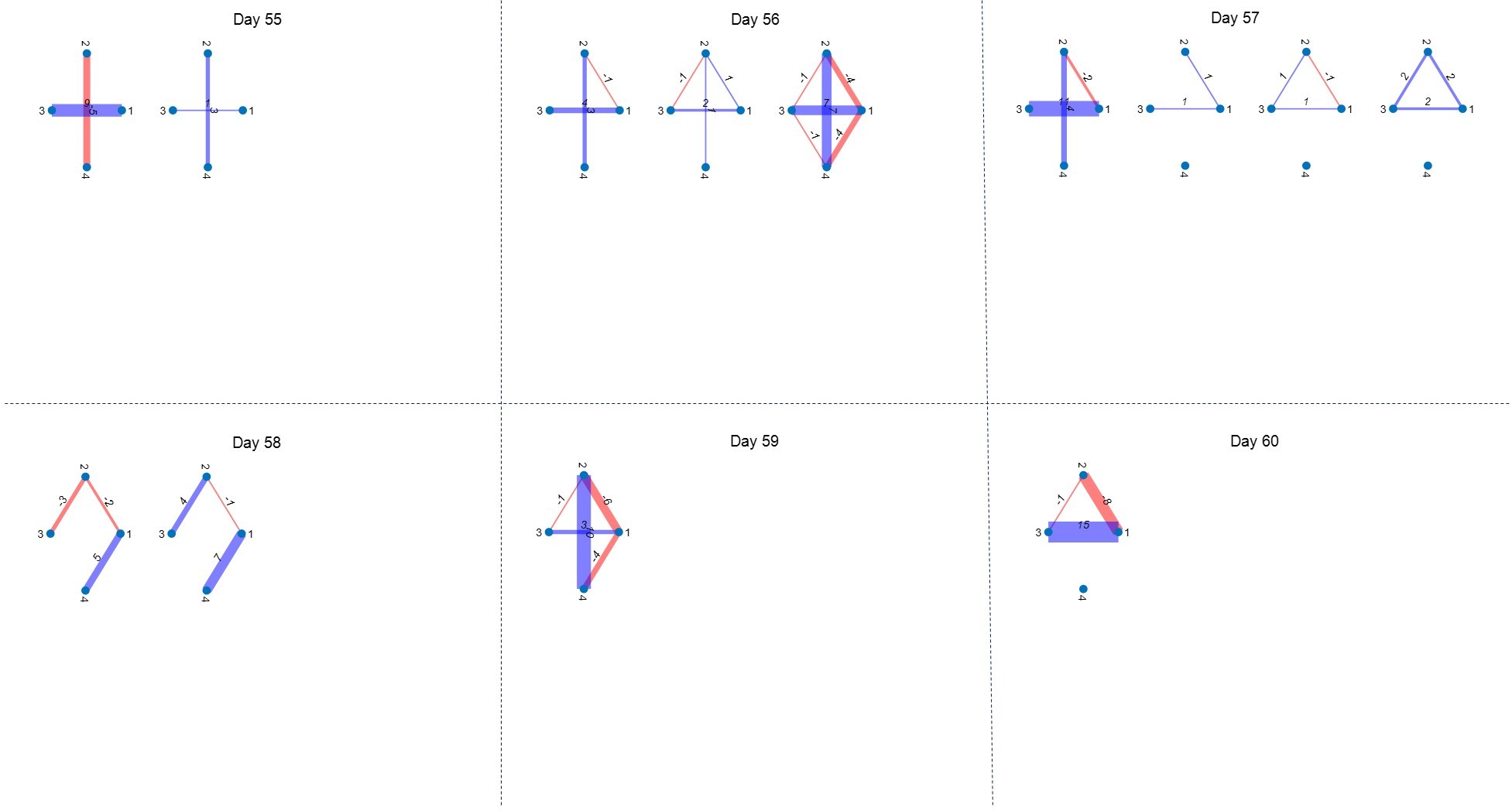
**


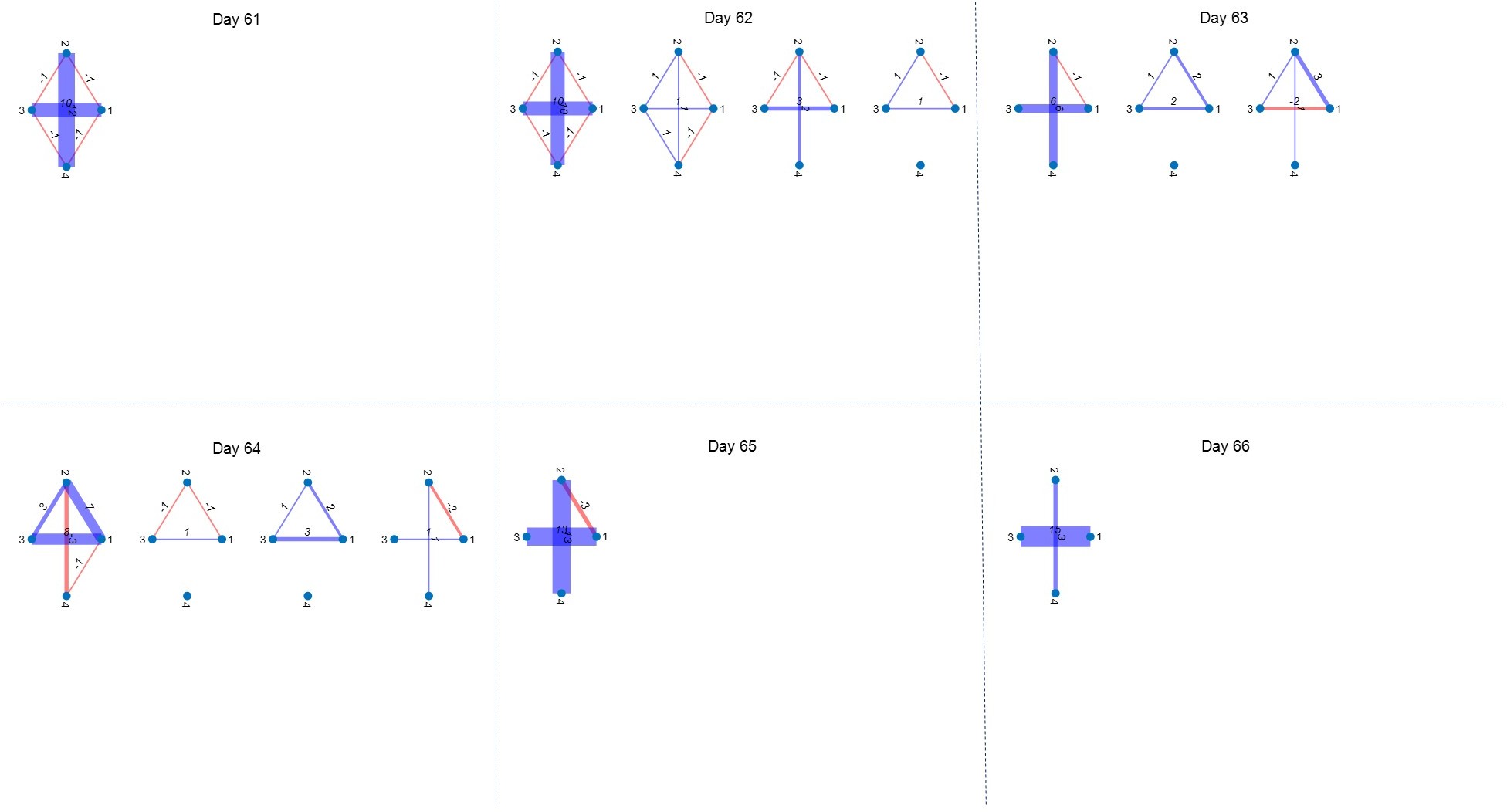


**
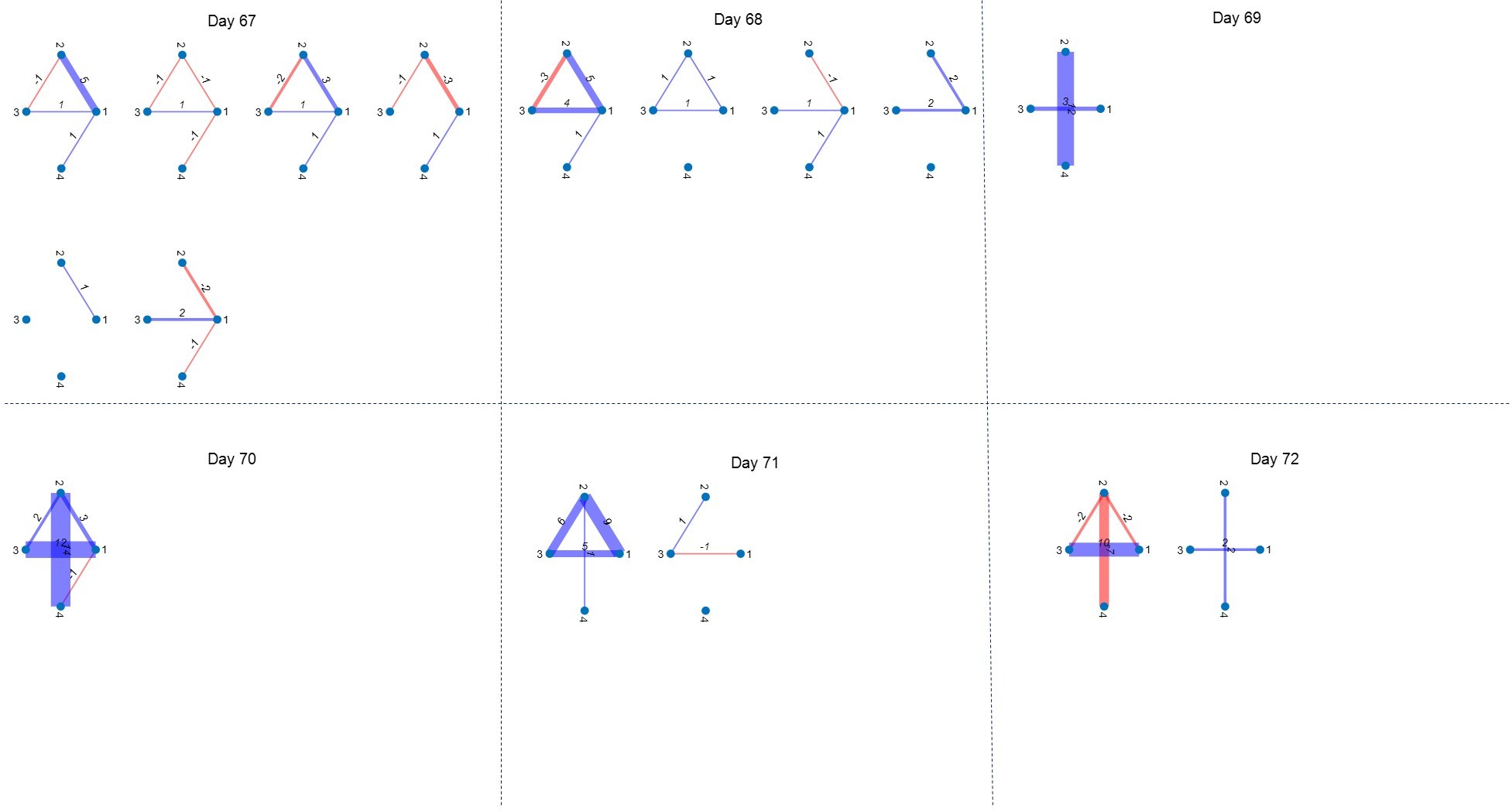
**


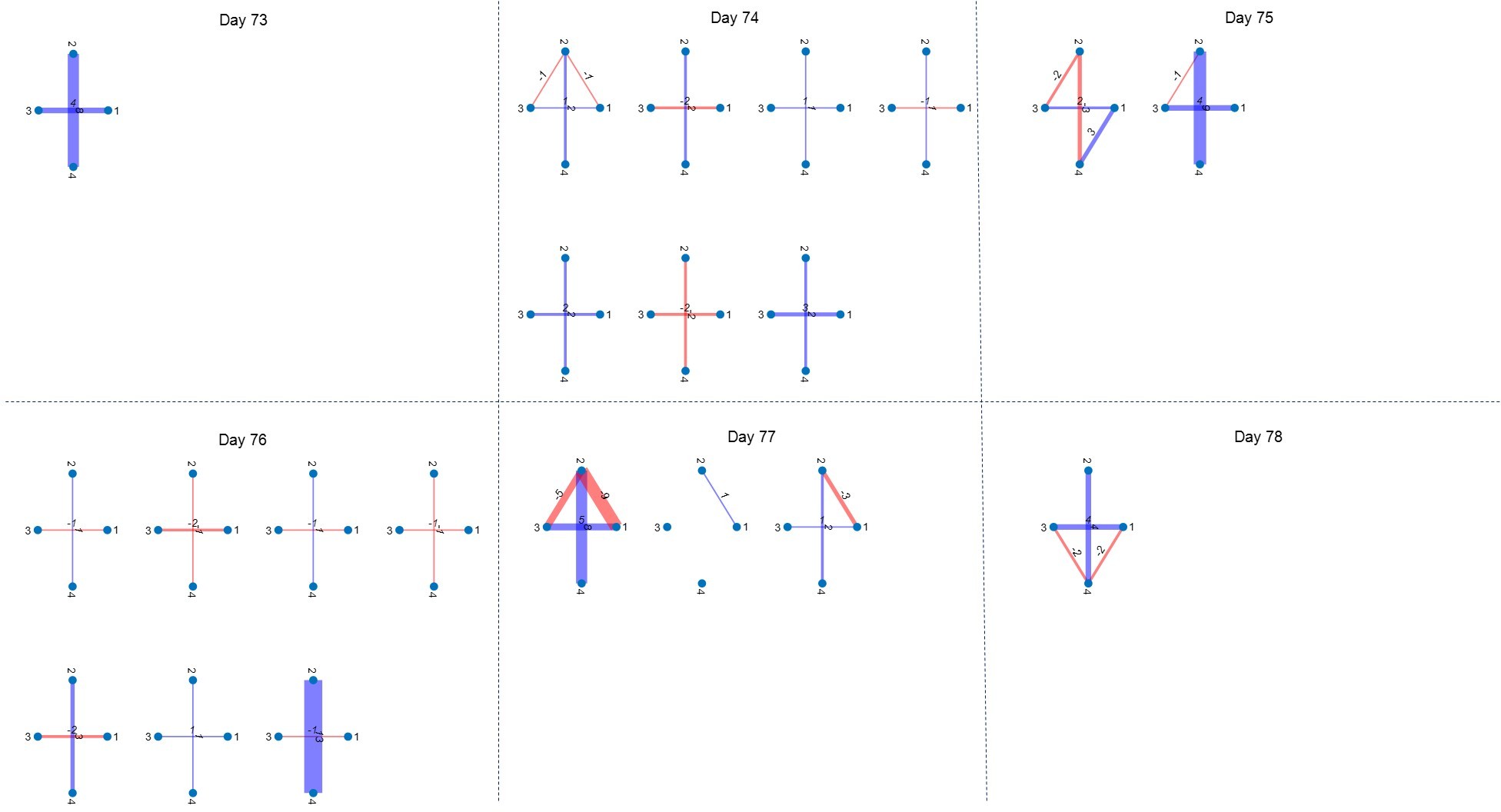


**
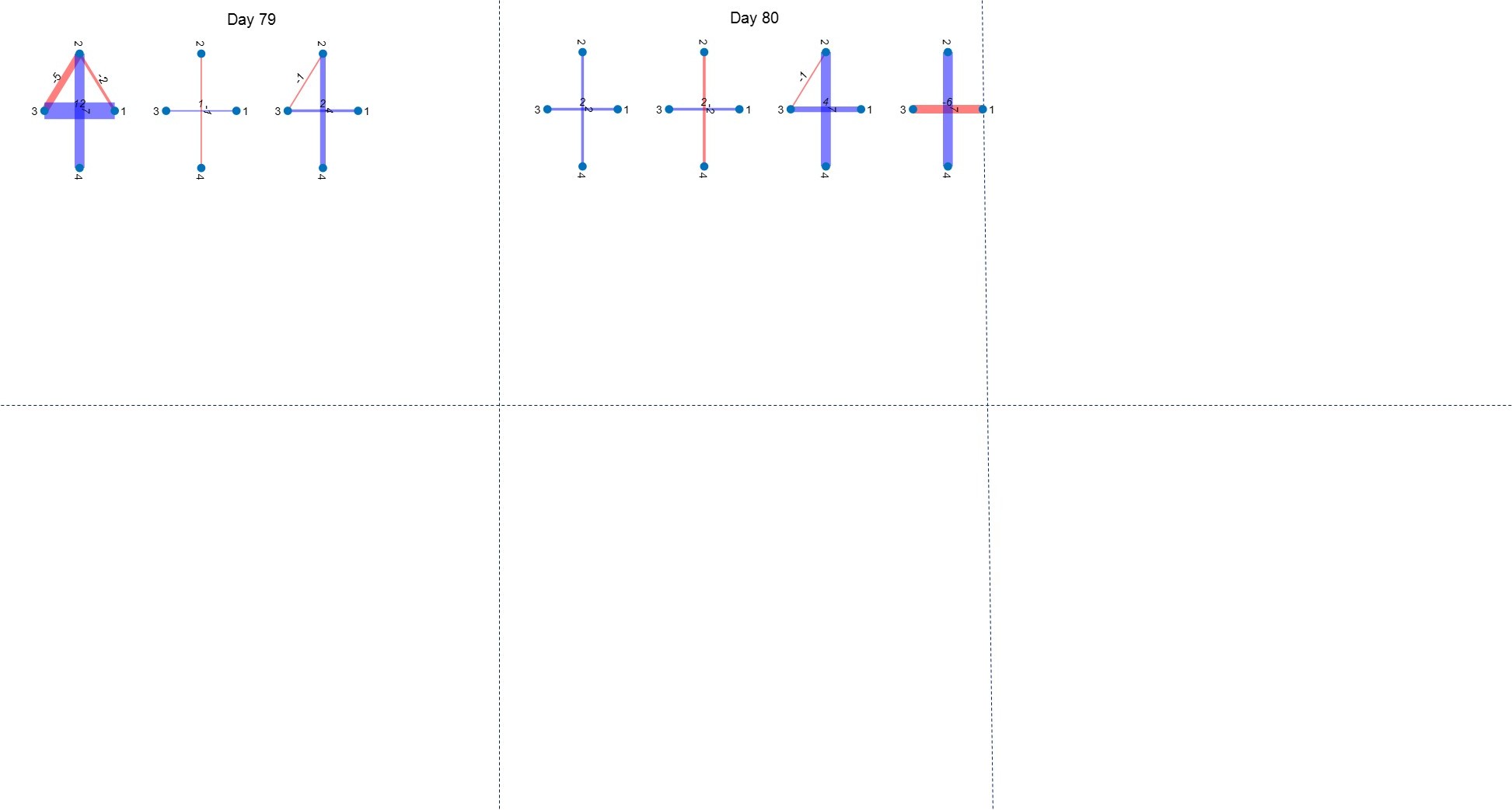
**

**Supp. Fig 4** Time aggregated networks of G2. The number accompanying the links indicate their weight. Red links represent aggressive interactions and blue ones affiliative interactions.

**
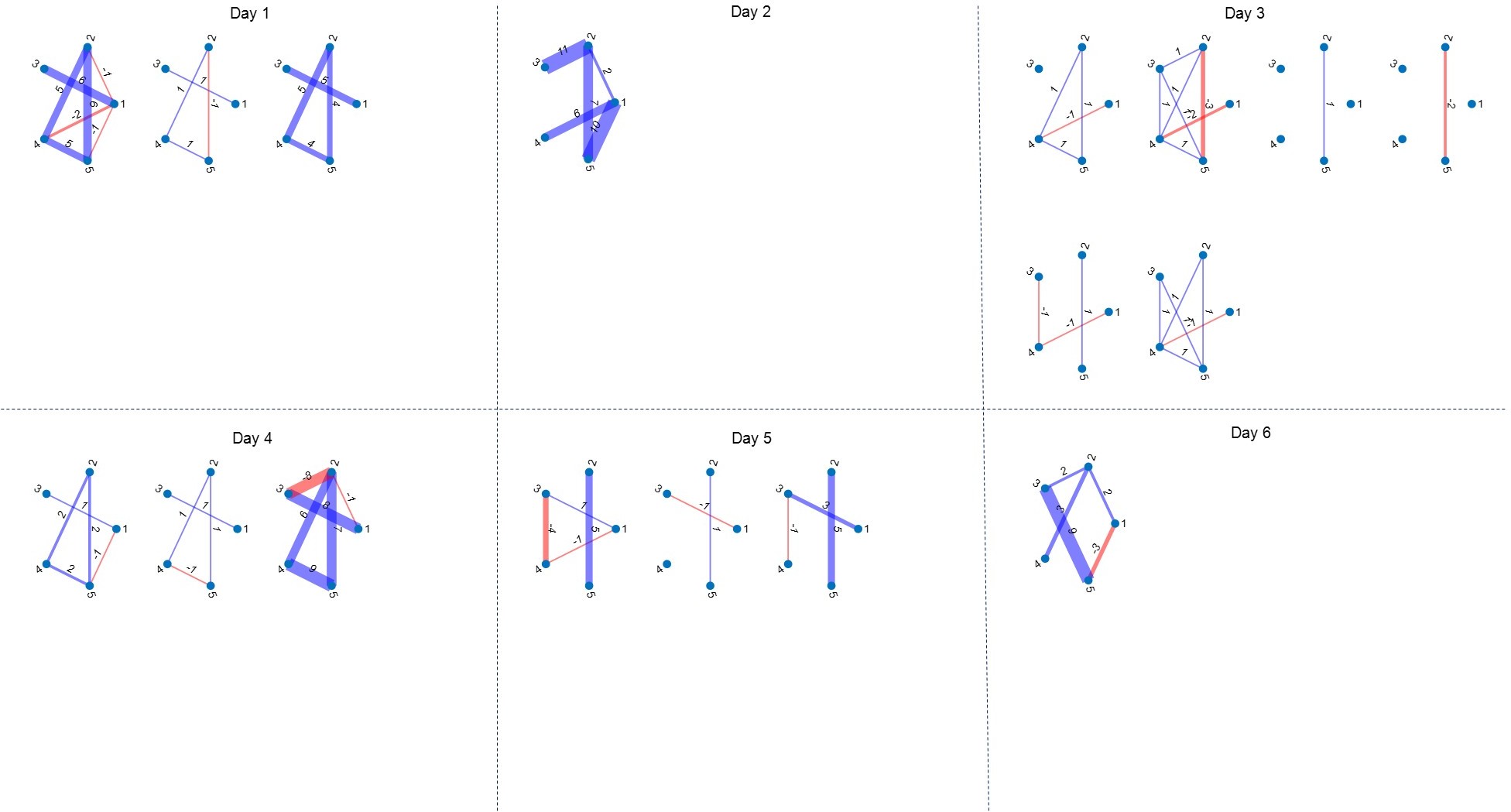
**

**
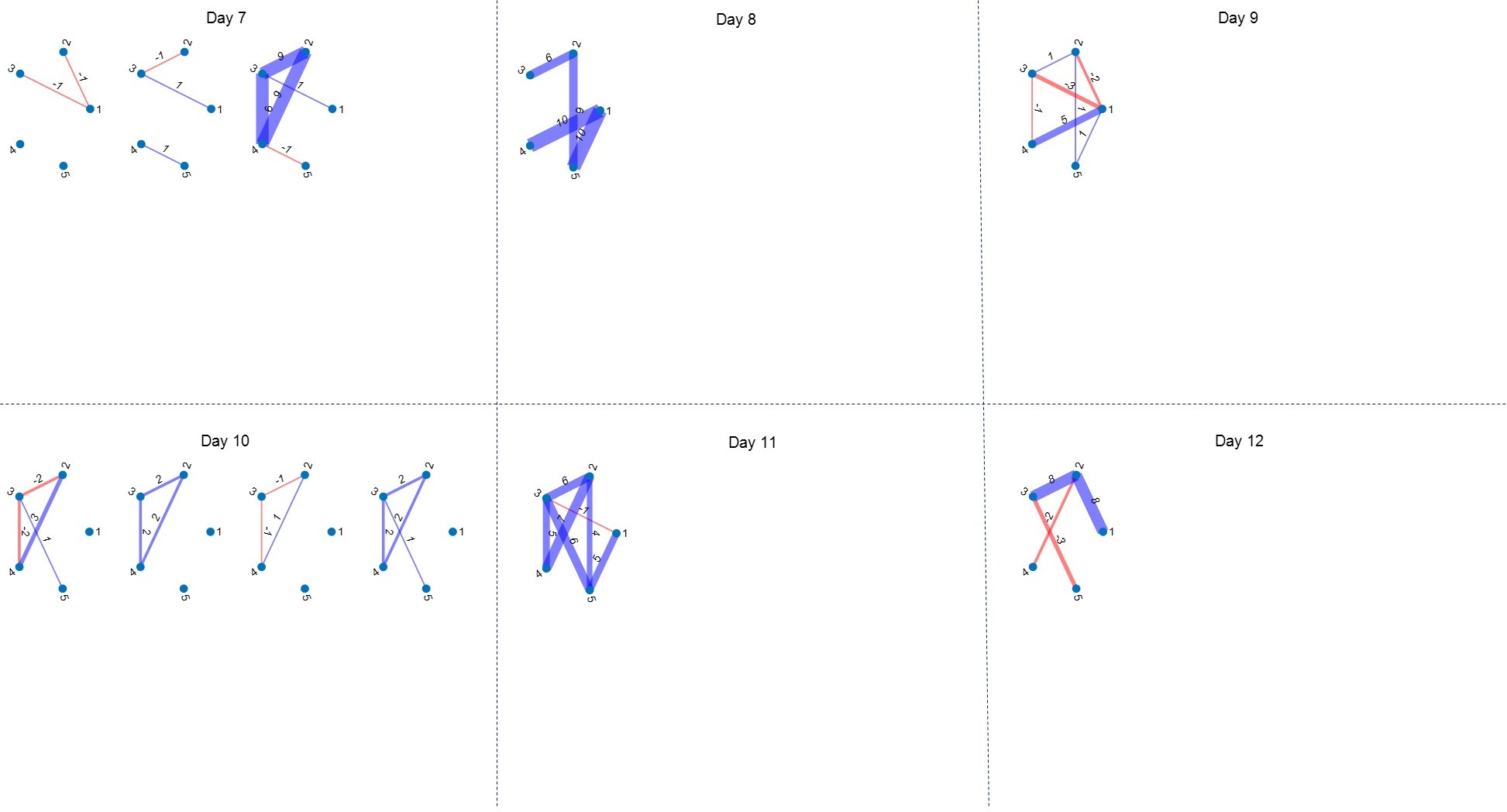
**


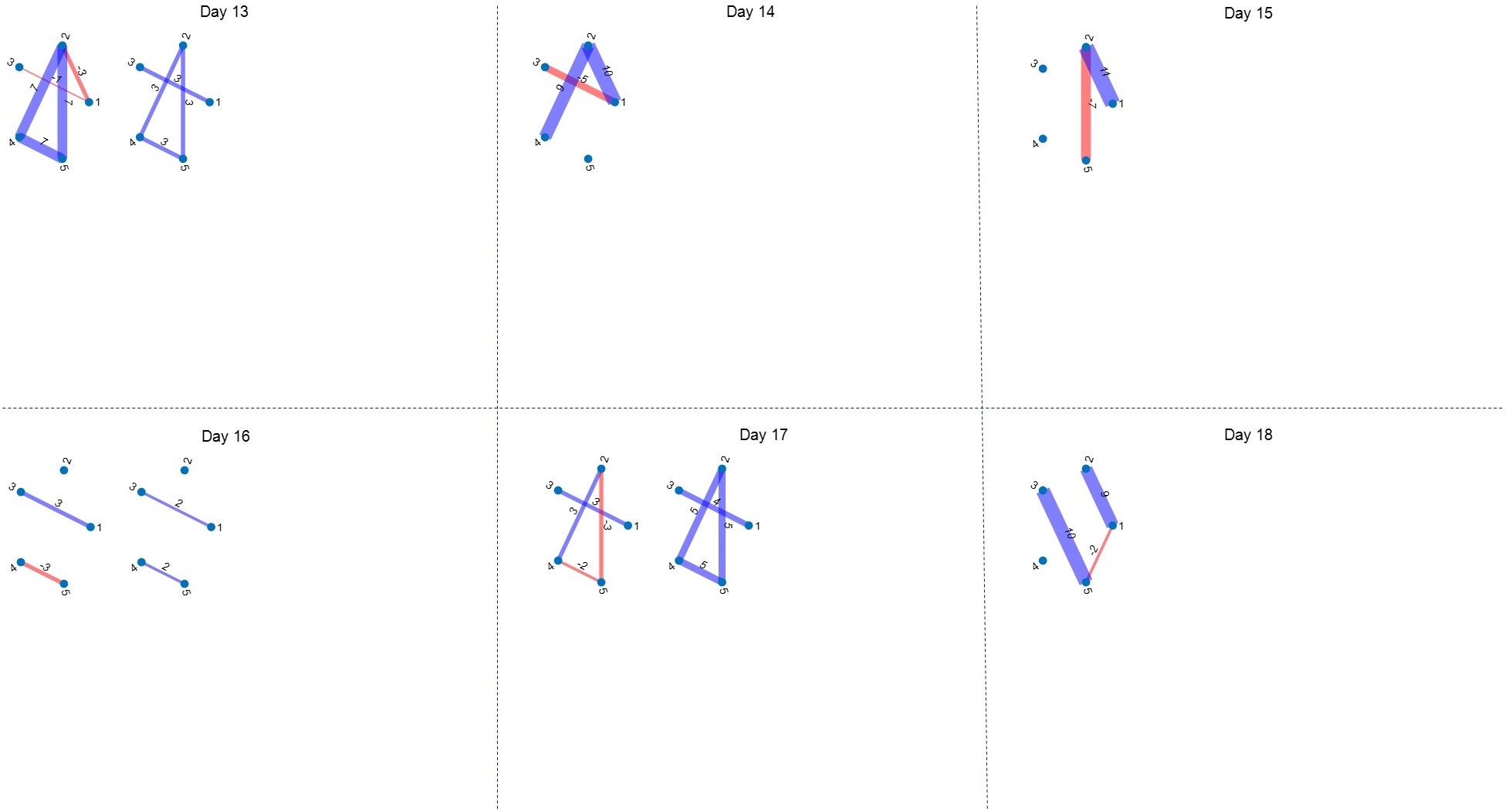


**
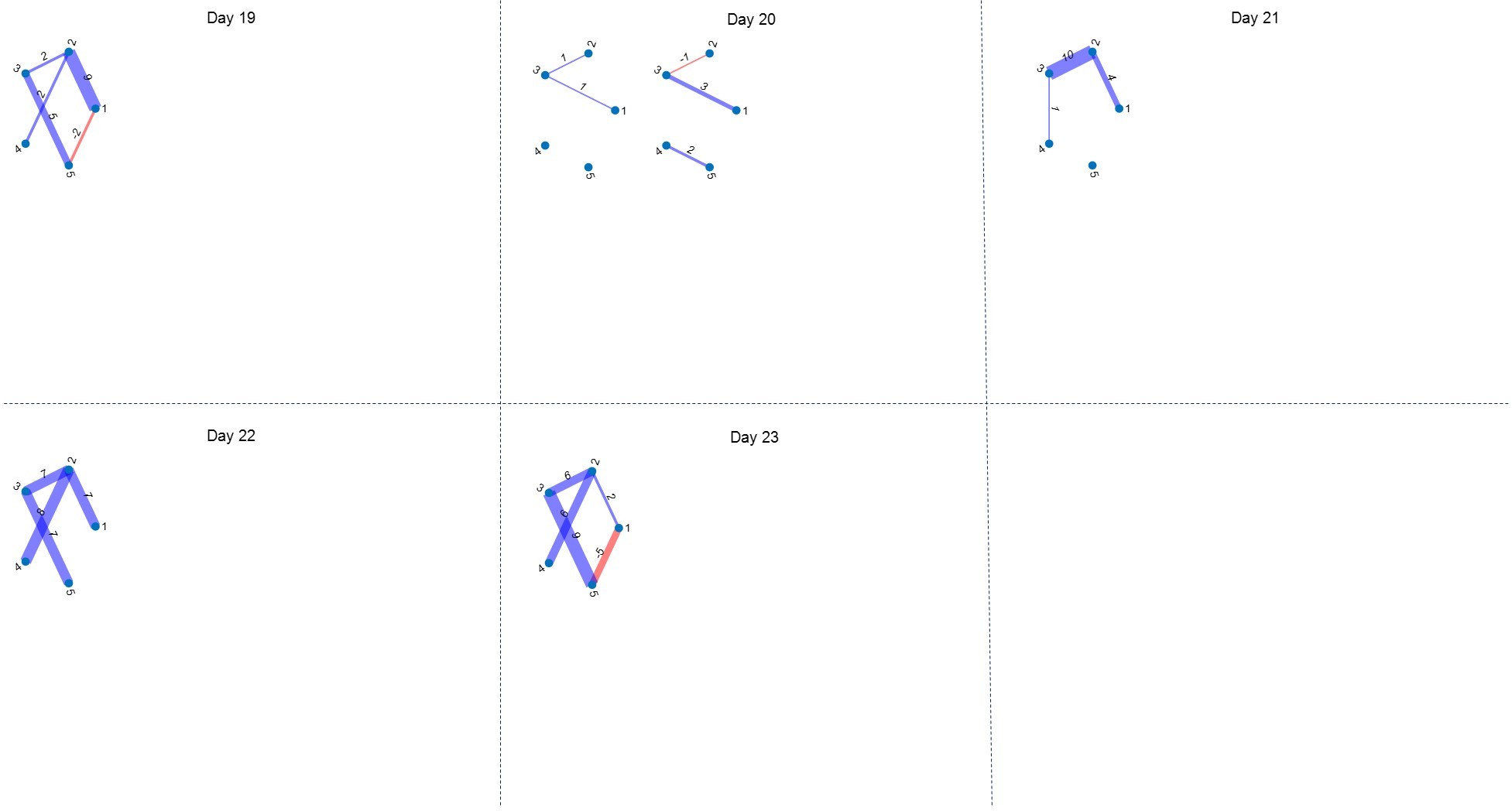
**
